# Deficiency of Microglial‐Derived Spp1 Exacerbates Age‐Related Memory Decline by Impairing Mitochondrial Complex I Function

**DOI:** 10.1111/acel.70378

**Published:** 2026-01-18

**Authors:** Meiling Wang, Yumin Chang, Aojie He, Jing Yang, Ang Li, Hongqin Wang, Kah‐Leong Lim, Xing Guo, Chengwu Zhang, Li Lu

**Affiliations:** ^1^ School of Basic Medical Sciences Shanxi Medical University Taiyuan Shanxi China; ^2^ Key Laboratory of Cellular Physiology, Ministry of Education Shanxi Medical University Taiyuan Shanxi China; ^3^ Key Laboratory of CNS Regeneration (Ministry of Education), Guangdong Key Laboratory of Non‐Human Primate Research, GHM Institute of CNS Regeneration Jinan University Guangzhou China; ^4^ Department of Neurosurgery The First Hospital of Shanxi Medical University Taiyuan China; ^5^ Lee Kong Chian School of Medicine Nanyang Technological University Singapore Singapore; ^6^ State Key Laboratory of Reproductive Medicine and Offspring Health Nanjing Medical University Nanjing China; ^7^ Department of Neurobiology, School of Basic Medical Sciences Nanjing Medical University Nanjing China

**Keywords:** age‐related memory decline, ATP, microglia, mitochondrial complex I, Spp1

## Abstract

Age‐related memory decline is a hallmark of brain aging and a primary risk factor for neurodegenerative disorders. Microglia play a crucial role in preserving memory function by maintaining brain homeostasis through phagocytosis, yet the specific mechanisms governing this protective function remain elusive. In the present study, we identified a population of Secreted Phosphoprotein 1 (Spp1)‐positive microglia in both aged mouse and human brains. To investigate the role of microglial Spp1 in aging, we generated microglia‐specific Spp1 knockout (Spp1‐cKO) mice. We demonstrate that Spp1 deficiency selectively precipitates memory deficits in aged mice, without affecting memory function in young mice, indicating an age‐dependent reliance on Spp1 signaling. Microglial phagocytic capacity positively correlates with Spp1 levels and is diminished by Spp1 deficiency. Mechanistically, Spp1 deficiency leads to the downregulation of the AKT/mitochondrial complex I pathway, thereby compromising microglial oxidative phosphorylation and function. Notably, microglia‐specific overexpression of Spp1 partially ameliorates the age‐related phenotypes induced by Spp1 deficiency. In conclusion, this study is the first to reveal the crucial role of microglial Spp1 in brain aging and to uncover its underlying mechanism, providing novel insights into age‐related memory decline.

## Graphical abstract

Microglia‐derived Spp1 regulates OXPHOS via the AKT/Complex I pathway to mitigate age‐related memory decline. (Graphical Abstract was created using BioRender).

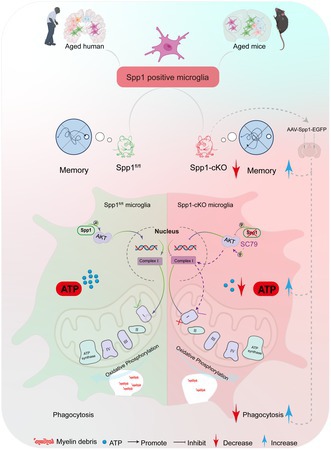

**Abbreviations**
AAV: adeno‐associated virus
AKT: protein kinase B
ATP: adenosine triphosphate
CNS: central nervous system
ECAR: extracellular acidification rate
EDTA: ethylenediaminetetraacetic acid
ELISA: enzyme‐linked immunosorbent assay
GSEA: gene set enrichment analysis
IBA1: ionized calcium‐binding adapter molecule 1
IF: immunofluorescence
LV: lentiviral vector
MACS: magnetic‐activated cell sorting
MG: microglia
MWM: morris water maze
OCR: oxygen consumption rate
OXPHOS: oxidative phosphorylation
ROS: reactive oxygen species
Spp1: secreted Phosphoprotein 1
TREM2: triggering receptor expressed on myeloid cells 2

## 1 Introduction

Brain aging constitutes a multifaceted physiological decline characterized by progressive memory loss and a heightened vulnerability to neurodegenerative disorders (e.g., Alzheimer's and Parkinson's diseases) (Shafqat et al. 2023). Among the various cellular mechanisms implicated in brain aging, microglia (MG) ‐ the resident immune cells of the central nervous system (CNS)—have garnered significant attention due to their crucial role in maintaining neural homeostasis through the clearance of pathological aggregates (e.g., amyloid‐β, α‐synuclein), microbial pathogens, and cellular debris (Rim et al. 2024). Recent advances in multi‐omics have identified key molecules, such as CD22 and TREM2, as critical regulators of microglial phagocytic function, and modulating their expression has been shown to help maintain microglial phagocytic function, thereby ameliorating age‐associated cognitive deficits (Pluvinage et al. 2019; Rachmian et al. 2024). Therefore, understanding how to restore or enhance microglial phagocytic function by modulating key molecules is crucial for the prevention and treatment of aging‐related phenotypes, yet the roles of these molecules remain poorly understood.

Emerging proteomic and single‐cell transcriptomic analyses reveal substantial diversification of microglia with age, including C1q^+^ populations linked to complement/proteostasis pathways and TREM2^+^ populations characterized by lipid–lysosomal and phagocytic remodeling (Rachmian et al. 2024; Scott‐Hewitt et al. 2024). However, aging brains likely harbor additional microglial subtypes with undefined functional roles that remain to be molecularly characterized. Age‐related decline in microglial phagocytic activity parallels a profound loss of metabolic plasticity, with dysfunction in oxidative phosphorylation (OXPHOS)—the principal bioenergetic engine driving phagocytosis—emerging as a key determinant of functional incompetence (Stoolman et al. 2024). Studies have shown that deficiencies in OXPHOS‐related proteins impair microglial phagocytic function and disrupt cognitive function in mice, suggesting metabolic modulation could be a potential therapeutic avenue (McElroy et al. 2020; Yuan et al. 2021). Specifically, studies in preclinical models reveal that genetic ablation of OXPHOS‐related components (e.g., Ndufs4 in Complex I) leads to microglial dysfunction, which in turn affects brain homeostasis (McElroy et al. 2020). Notably, rescue experiments show that pharmacological enhancement of mitochondrial OXPHOS—through NAD^+^ precursors (e.g., nicotinamide riboside) or sodium rutin—restores microglial phagocytic function and reverses memory deficits (Hou et al. 2018; Pan et al. 2019). Thus, deciphering how to restore microglial subset‐specific bioenergetic states through OXPHOS modulation emerges as a pivotal strategy for preserving cognitive integrity.

Secreted Phosphoprotein 1 (Spp1), a multifunctional protein encoded by the Spp1 gene, is highly expressed in the CNS (Yim et al. 2022). Spp1 acts as a pivotal damage sensor in the CNS, triggering responses upon sensing cellular damage or disruptions in homeostasis (Yim et al. 2022). Although high Spp1 levels in various pathological conditions are often considered pro‐inflammatory and pathogenic, recent studies reveal its dual role in disease regulation—acting both as a neuroprotective agent that reduces inflammation and promotes tissue repair, and as a factor that drives inflammation and disease progression (Lin et al. 2023). This “double‐edged sword” nature suggests that the role of Spp1 in CNS diseases depends on the disease context, the cell type‐specific expression, and the dynamic balance of its interaction network (Lin et al. 2023). Notably, MG‐derived Spp1 plays a critical role in shaping brain function during both fetal and developmental stages (Lawrence et al. 2024; Shen et al. 2022). Moreover, recent studies utilizing single‐cell RNA sequencing (scRNA‐seq) have revealed that Spp1 expression in MG is altered in various CNS disorders, including Alzheimer's disease (AD), major depressive disorder, and acute ischemic stroke (Scheepstra et al. 2023; Spitzer et al. 2022; Zhou et al. 2020). These findings suggest that Spp1, particularly when derived from MG, plays a crucial role in CNS function. However, the role of MG‐derived Spp1 in maintaining memory function and its underlying mechanisms remain to be fully elucidated.

In the present study, we identified a distinct subpopulation of Spp1‐positive MG in both aged mouse and human brains. Functionally, we demonstrated that MG‐specific deficiency of Spp1 selectively impaired memory in aged mice, without affecting memory function in young mice. Moreover, Spp1 expression positively correlated with the phagocytic activity of MG, and its knockdown impaired this function. Mechanistically, we revealed that Spp1 deficiency inhibited the AKT/mitochondrial complex I signaling pathway, thereby compromising OXPHOS in MG. Importantly, administration of the AKT agonist SC79 restored MG function and reversed the memory deficits induced by Spp1 deficiency. Finally, we demonstrated that MG‐specific overexpression of Spp1 was sufficient to rescue the impairments in cellular function (phagocytosis and ATP production) and memory driven by Spp1 deficiency. Collectively, this study provides the first evidence that Spp1 deficiency exacerbated memory decline by disrupting the AKT/mitochondrial complex I pathway‐mediated OXPHOS, while MG‐specific overexpression of Spp1 restored these phenotypes. These findings suggest that targeting Spp1 may serve as a potential therapeutic strategy for combating age‐related memory decline.

## 2 Results

### 2.1 Microglia‐Derived Spp1 Deficiency Exacerbated Age‐Related Memory Decline in Mice

**FIGURE 1:**
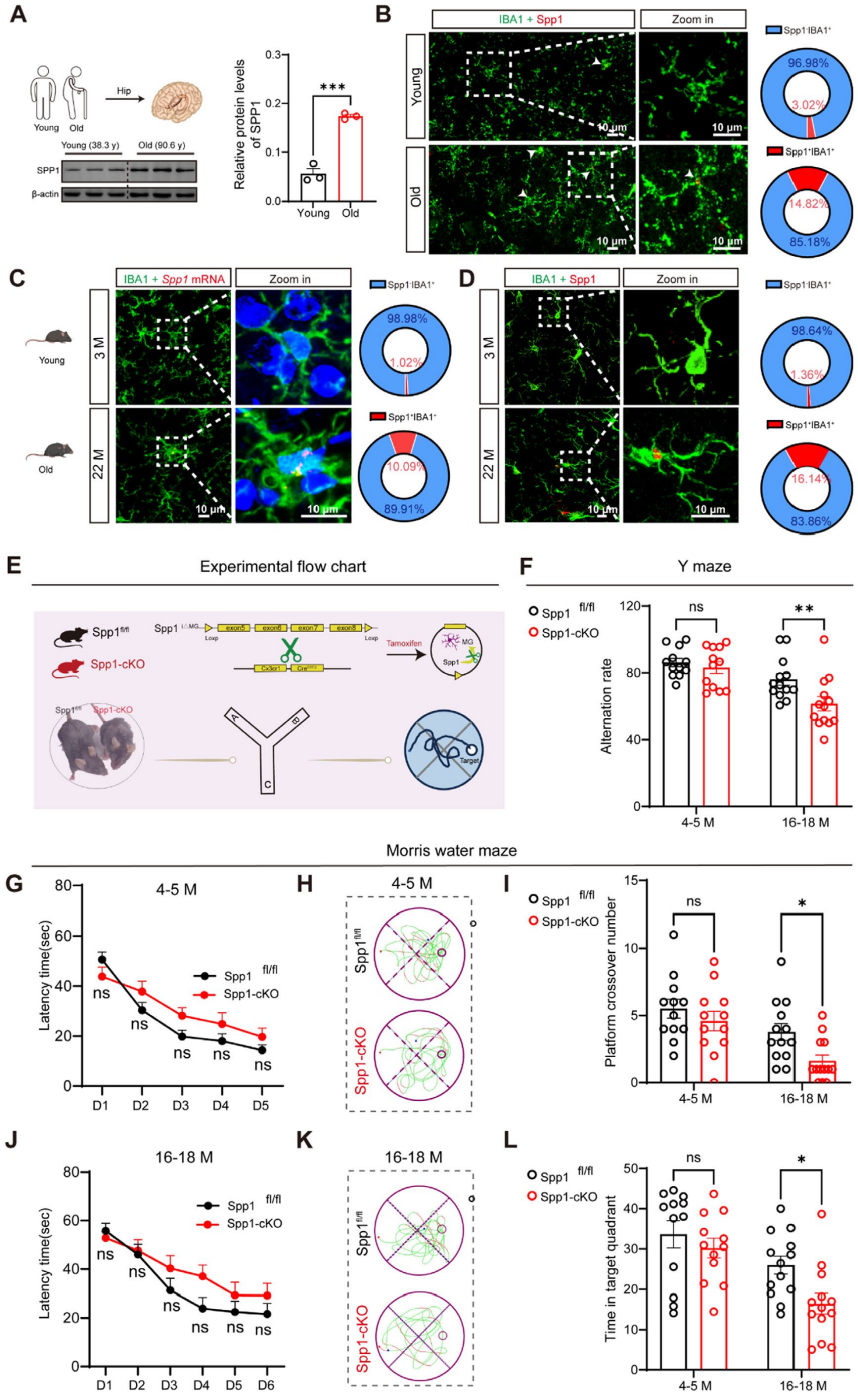
Microglial Spp1 deficiency led to age‐dependent memory deficits. (A) Western blot analysis of Spp1 protein in the hippocampus of young (31–43 years; mean 38.3) and old humans (88–95 years; mean 90.6) (*n =* 3 per group). (B) Representative images showing the co‐localization of Spp1 and the microglial marker (IBA1) in brain sections from young and aged humans. Dashed white boxes indicate magnified images of individual cells, and donut chart point to IBA1^+^Spp1^+/−^ cells, Scale bars = 10 μm (*n =* 3 per group). (C) Representative images showing the co‐localization of Spp1 mRNA and the microglial marker (IBA1) in brain sections from young (3 M) and aged (22 M) mice. Dashed white boxes indicate magnified images of individual cells, and donut chart indicate the proportion IBA1^+^Spp1 mRNA^+/−^ cells, Scale bars = 10 μm (*n =* 3 per group). (D) Representative images showing the co‐localization of Spp1 and the microglial marker (IBA1) in brain sections from young (3 M), and aged (22 M) mice. Dashed white boxes indicate magnified images of individual cells, and donut chart point to IBA1^+^Spp1^+/−^ cells, Scale bars = 10 μm (*n =* 3 per group). (E) Schematic diagram of the experimental design. (F) Percentage of correct alternations in the Y‐maze test of adult (*n =* 12) and aged (*n =* 13) Spp1^fl/fl^ mice and Spp1‐cKO mice. (G/J) Learning curves during Morris Water Maze (MWM) training for adult (G) (*n =* 12) and aged (J) (*n =* 13) Spp1^fl/fl^ and Spp1‐cKO mice, measured by latency to find the platform. (H/K) Representative path plots showing the search patterns of adult (H) and aged (K) Spp1^fl/fl^ and Spp1‐cKO mice during the MWM probe trial. (I) Number of platform crossings during the MWM probe trial of adult (*n =* 12) and aged (*n =* 13) Spp1^fl/fl^ mice and Spp1‐cKO mice. (L) Time spent in the target quadrant during the MWM probe trial of adult (*n =* 12) and aged (*n =* 13) Spp1^fl/fl^ mice and Spp1‐cKO mice. Data are presented as the mean ± standard error of the mean (SEM). Two‐way repeated measures ANOVA was used to compare the latency to platform acquisition during the learning curves (G/J). Other data were analyzed using unpaired two‐tailed *t*‐tests. *p < 0.05, **p < 0.01, ***p < 0.001, ns = no significant difference. Figure 1E was created using BioRender.

During brain aging, the accumulation of dysfunctional cells and abnormal metabolites leads to an imbalance in the brain microenvironment, while the transcription of genes exhibits dynamic plasticity to counteract these perturbations through rapid adaptive responses (Rim et al. 2024). To verify Spp1 expression trends in our experimental model, qPCR and Western blot analyses confirmed that both Spp1 mRNA and protein levels were significantly elevated in the hippocampus of aged (22‐month‐old) mice compared to young (3‐month‐old) mice (Figure S1A,B). Furthermore, Spp1 expression was significantly elevated in the hippocampus of aged individuals (average age 90.6 years) compared to that of adult individuals (average age 38.3 years) (Figure 1A). To identify the cell types that preferentially express Spp1, scRNA‐seq and immunofluorescence (IF) staining analyses were conducted. Based on the annotation of scRNA‐seq data (GSE129788) from aged mouse brains, we found that among the seven primary cell types, MG exhibited the highest expression of Spp1 (Figure S1C). As shown in Figure S1D–K, Spp1‐positive signal was predominantly observed in IBA1 positive MG, with undetectable expression in oligodendrocytes (Olig2‐positive), neurons (NeuN‐positive), and astrocytes (GFAP‐positive).

To further verify age‐related changes in Spp1 specifically in microglia across species, we analyzed additional transcriptomic datasets. In mice (GSE156762), *Spp1* expression was significantly upregulated in microglia from aged (23‐month‐old) compared with young mice (6‐month‐old) (Figure S1L). Similarly, in human samples (GSE99074), *SPP1* expression was robustly increased in aged (84–85 years; mean 84.7) compared with young (31–43 years; mean 38.3) individuals (Figure S1M). Next, through multiplex fluorescent immunohistochemical staining (mIHC), we found that the proportion of Spp1^+^ IBA1^+^ double‐positive MG was increased in the aged individuals' brains compared to that of adult ones, with the proportion 14.82% and 3.02% in aged and adult brains, respectively (Figure 1B). Moreover, fluorescence in situ hybridization (FISH) results (Figure 1C) showed that the proportion of Spp1 mRNA^+^ IBA1^+^ double‐positive MG was higher in the brains of aged mice compared to those of young mice, accounting for 10.09% and 1.02%, respectively. To confirm Spp1 expression at the protein level, we conducted double IF staining. This study showed that the proportion of Spp1^+^ IBA1^+^ double‐positive MG in the brains of aged and young mice was 16.14% and 1.36%, respectively (Figure 1D). These results indicate that expression of Spp1 is enhanced in aged brain, and MG are the type of cells that preferentially expressed Spp1.

After observing the expression profile of MG‐derived Spp1 in the aged brain, we sought to determine its role in aging. To this end, we generated inducible MG‐specific Spp1 conditional knockout (cKO) mice using the Cre‐LoxP system (Cx3cr1^CreERT2^; Spp1^fl/fl^, referred to as Spp1‐cKO) (Figure 1E). Spp1 was efficiently knocked out in aged Spp1‐cKO mice without compromising microglial density (Figure S2A–D). To investigate the impact of microglial Spp1 deficiency on cognition, we assessed both young (4–5 months) and aged (16–18 months) mice. Behaviorally, while young (4–5 months) Spp1‐cKO mice exhibited normal cognition (Figure 1F,I,L), aged (16–18 months) Spp1‐cKO mice displayed significant deficits. Specifically, aged Spp1‐cKO showed reduced alternation in the Y‐maze (Figure 1F) and impaired spatial memory in the Morris water maze probe trial (Figure 1I,L), despite preserved learning curves (Figure 1G,J). These results demonstrate that Spp1 deficiency selectively impairs memory in the aging brain. To further understand the molecular context, we quantified hippocampal p53 levels and found a significant upregulation in Spp1‐cKO mice compared with controls (Figure S2E). Next, we measured pro‐inflammatory cytokines (IL‐1β, IL‐6, TNF‐α) in the hippocampus and observed marked increases in all three factors in Spp1‐cKO mice (Figure S2F–H). To confirm that this pro‐inflammatory phenotype is intrinsic to microglia, we further assessed cytokine secretion in primary microglia isolated from Spp1^fl/fl^ and Spp1‐cKO mice. Consistent with the in vivo findings, Spp1‐deficient microglia released significantly higher levels of TNF‐α, IL‐6, and IL‐1β compared to controls (Figure S2I–K). These elevated factors serve as important indicators of age‐related memory decline, consistent with the behavioral deficits observed in Spp1‐deficient mice.

### 2.2 Spp1 Deficiency Impaired Phagocytic Capacity of Microglia in Aged Mice

**FIGURE 2:**
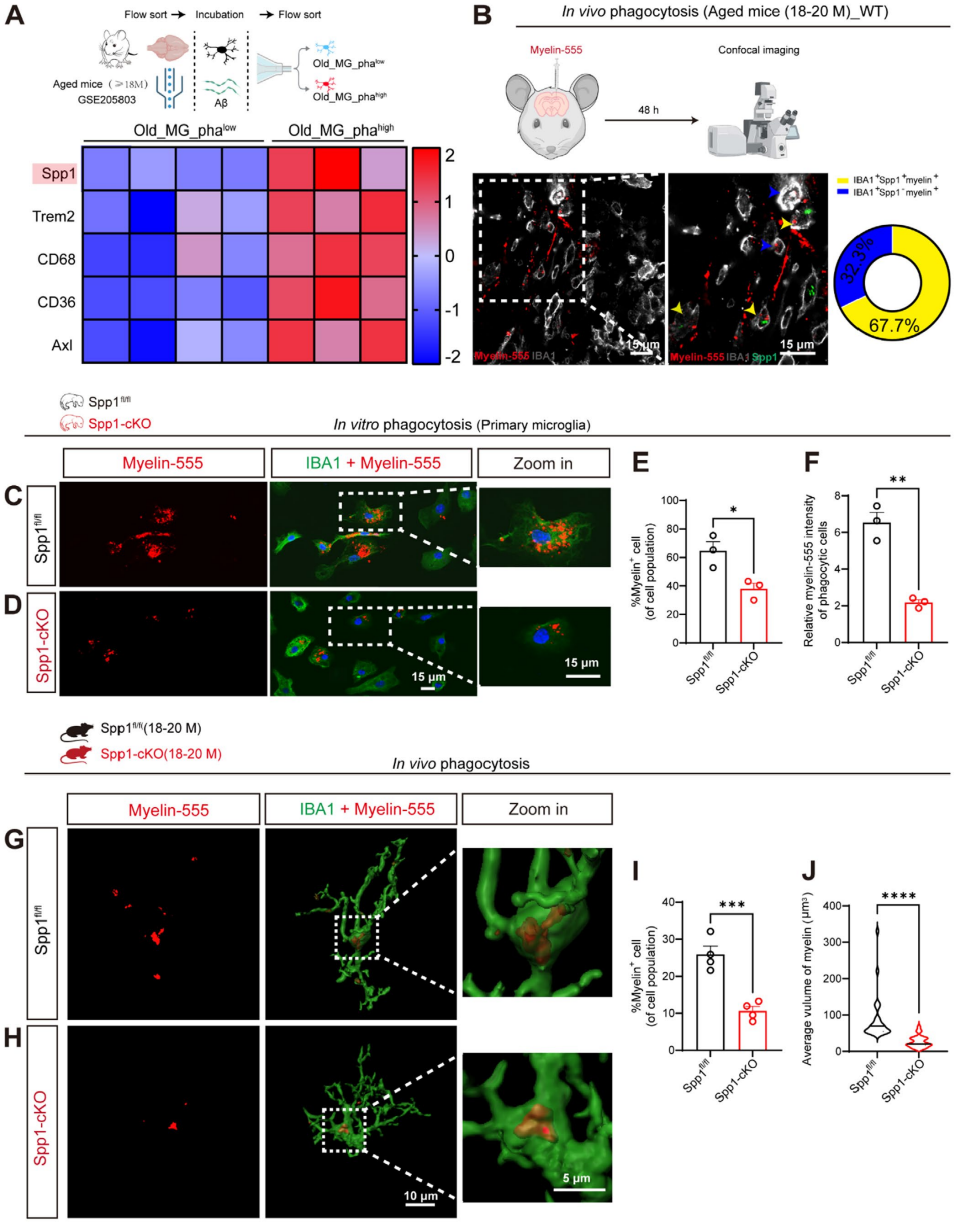
Deficiency of Spp1 impaired microglial phagocytic function. (A) Heatmap showing differentially expressed genes (DEGs) between two groups of aged MG with high (*n* = 3) and low (*n* = 4) phagocytic activity. Red indicates upregulated genes, while blue indicates downregulated genes. (B) Representative images of microglial uptake of myelin‐555 in aged mice. Yellow arrows indicate Spp1‐positive microglia phagocytosing myelin‐555; blue arrows indicate Spp1‐negative microglia phagocytosing myelin‐555. Scale bars = 15 μm (*n =* 6 mice per group). (C, D) Representative images showing phagocytosis of myelin‐555 by primary microglia isolated from postnatal day 3 (P3) Spp1^fl/fl^ and Spp1‐cKO mice; white dashed lines indicating magnified images of individual MG. Scale bars = 15 μm. (E) %Myelin^+^cell (of cell population) = number of phagocytic cells/total cell count (*n =* 3 per group). (F) Relative myelin‐555 intensity of phagocytic cells = total fluorescence intensity/total number of phagocytic cells (*n =* 3 per group). (G, H) Myelin‐555 was stereotactically injected into the hippocampus of aged Spp1^fl/fl^ and Spp1‐cKO mice to assess MG phagocytosis. Scale bars, 10 μm (low magnification) and 5 μm (high magnification). (I) Proportion of myelin^+^ MG (*n =* 4 per group). (J) Analysis of myelin volume within MG (*n =* 4 per group). Data are presented as the mean ± standard error of the mean (SEM). Data were analyzed by unpaired two‐tailed *t*‐tests. *p < 0.05, **p < 0.01, ***p < 0.001. Mouse/Cell pattern was created using BioRender.

During aging, MG maintain brain homeostasis by phagocytosing waste products accumulated in the brain, such as Aβ and myelin debris (Marschallinger et al. 2020; Rodrigue et al. 2009). Given the observed phenotype of Spp1‐cKO mice, the impact of Spp1 deficiency on the phagocytic capacity of MG was further investigated. Firstly, we analyzed RNA‐Seq data from the Gene Expression Omnibus (GEO) database and revealed that MG populations with higher levels of Spp1 exhibited enhanced phagocytic capacity compared to those with lower Spp1 expression (Figure 2A). To confirm this observation, fluorescence‐labeled myelin debris (myelin‐555) was stereotactically injected into the hippocampus of aged wild‐type mice. Spp1‐positive MG phagocytosed more myelin‐555 than Spp1‐negative MG (Figure 2B). These results underscore the critical role of Spp1 in maintaining the phagocytic function of MG in aged mice.

To further assess the impact of Spp1 knockout on MG phagocytosis, MG were isolated from Spp1‐cKO and Spp1^fl/fl^ mice, and then exposed to myelin‐555 to evaluate their phagocytic capacity (Figure 2C,D). Spp1‐KO MG showed a significantly reduced number of myelin‐555–positive cells compared with MG from Spp1^fl/fl^ mice (Figure 2E). Moreover, the fluorescence intensity of myelin‐555 in Spp1‐KO MG was markedly weaker than that in MG from Spp1^fl/fl^ mice (Figure 2F). Consistent with these findings in primary cells, BV2 cells transfected with lentivirus (LV)‐*Spp1*‐shRNA also exhibited compromised phagocytic capacity (Figure S3E,F). Meanwhile, Spp1‐deficient microglia also exhibited significantly reduced phagocytosis of Aβ oligomers (Figure S3G,H), indicating a broad impairment in phagocytic function. To further confirm the impact of Spp1 on MG phagocytic function in vivo, myelin‐555 was stereotactically injected into the hippocampus of aged Spp1‐cKO and aged Spp1^fl/fl^ mice. The number of myelin‐555‐positive MG in the brains of Spp1‐cKO mice was significantly lower than in Spp1^fl/fl^ mice (Figure 2I). Furthermore, the volume of myelin engulfed by MG in the brains of Spp1‐cKO mice was significantly reduced compared to that in Spp1^fl/fl^ mice (Figure 2J). These findings strongly suggest that Spp1 is essential for modulating the phagocytic capacity of MG.

### 2.3 Deficiency of Spp1 in Microglia Disrupted Oxidative Phosphorylation

**FIGURE 3:**
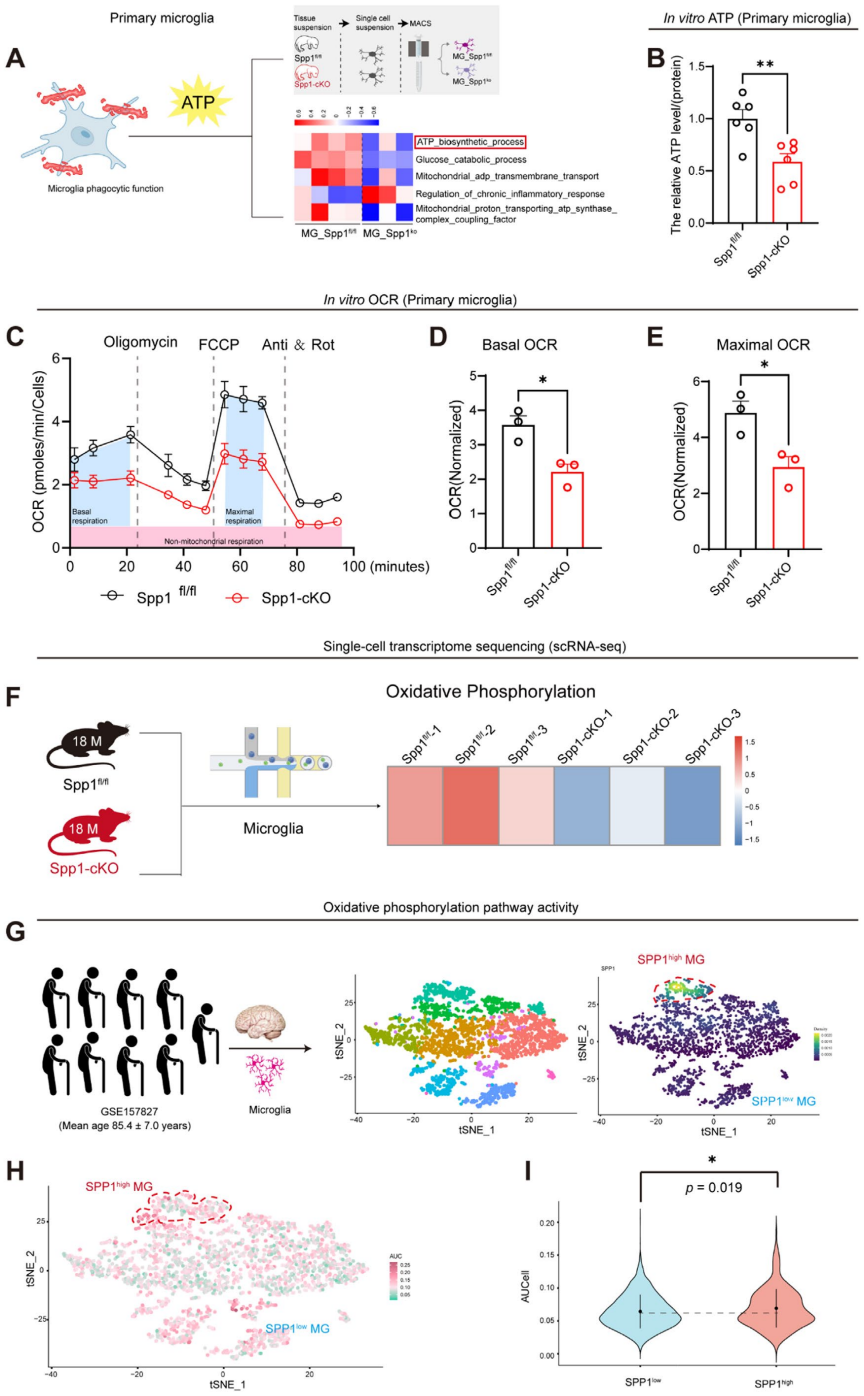
Deficiency of Spp1 disrupted microglia oxidative phosphorylation. (A) Heatmap of GSVA analysis comparing gene set enrichment between primary microglia isolated from postnatal day 3 (P3) Spp1^fl/fl^ and Spp1‐cKO mice; blue indicates downregulation, and red indicates upregulation. (B) Quantification of ATP levels in primary microglia isolated from postnatal day 3 (P3) Spp1^fl/fl^ and Spp1‐cKO mice (*n =* 6 per group). (C) Measurement of Seahorse XF mitochondrial stress OCR in primary microglia from postnatal day 3 (P3) Spp1^fl/fl^ and Spp1‐cKO mice. (D) Basal OCR (*n =* 3 per group). (E) Maximum OCR (*n =* 3 per group). (F) Heatmap of GSVA pathway scores showing significant downregulation of Oxidative Phosphorylation in the microglial cluster of aged (18‐month‐old) Spp1‐cKO mice compared to controls. (G) Subsets of high SPP1‐expressing (SPP1^high^ MG) and low SPP1‐expressing (SPP1^low^ MG) in aged human brains (GSE157827). (H) Oxidative phosphorylation pathway scores in SPP1^high^ and SPP1^low^ MG in aged human brains. (I) Violin plot showing AUCell scores for SPP1^high^ MG and SPP1^low^ MG cells. Data are presented as the mean ± standard error of the mean (SEM). Statistical significance was determined using unpaired two‐tailed *t*‐tests for (B, D, E) and Wilcoxon rank‐sum test for (I). FCCP, Carbonyl cyanide 4‐trifluoromethoxyphenylhydrazone; Anti & Rot, Antimycin A and Rotenone; 2‐DG, 2‐Deoxy‐D‐glucose. *p < 0.05, **p < 0.01, ns = no significant difference. Human/Mouse/Cell pattern was created using BioRender.

Energy homeostasis is essential for maintaining MG phagocytic function (Bennett and Liddelow 2019). Given that MG‐specific knockout of Spp1 compromised their phagocytic function, we explored the underlying mechanism by transcriptome analysis utilizing MG isolated from Spp1‐cKO and Spp1^fl/fl^ mice. To exclude potential viability artifacts, we first confirmed that Spp1 deficiency did not affect cell survival (Figure S4A). The results showed that, compared to MG isolated from Spp1^fl/fl^ mice, MG from Spp1‐cKO mice exhibited a significant downregulation of the ATP biosynthetic process (Figure 3A). Subsequently, ATP levels in primary cultured MG derived from Spp1^fl/fl^ and Spp1‐cKO mice were assessed. As shown in Figure 3B, the ATP level in MG from Spp1‐cKO mice was significantly lower than that of Spp1^fl/fl^ mice. ATP production occurs primarily through two pathways: OXPHOS and glycolysis (Kim et al. 2024). To determine which pathway was affected, DEGs from RNA‐seq data of MG isolated from Spp1‐cKO and Spp1^fl/fl^ mice were analyzed. Gene set enrichment analysis (GSEA) revealed that the OXPHOS pathway was significantly down‐regulated in MG isolated from Spp1‐cKO mice compared to those from Spp1^fl/fl^ mice (Figure S4B). In contrast, genes involved in glycolysis showed no significant changes (Figure S4C–J). To further validate these findings, the Seahorse XF analyzer, a widely used tool for assessing cellular energy metabolism, was employed. The results showed that both the basal and maximum oxygen consumption rates (OCR) of MG isolated from Spp1‐cKO mice were significantly lower than those of control MG (Figure 3C–E). However, no significant differences were observed in the basal or maximum extracellular acidification rate (ECAR) between the two groups (Figure S4K–M). These findings suggest that Spp1 regulates microglial ATP production through OXPHOS, rather than glycolysis.

To rigorously validate whether this metabolic defect persists in vivo within the aging microenvironment, we performed scRNA‐seq on hippocampal tissues from 18‐month‐old Spp1‐cKO and Spp1^fl/fl^ mice (Figure 3F). Consistent with ou*r* in vitro findings, Gene Set Variation Analysis (GSVA) of the microglial cluster revealed a specific and significant downregulation of Oxidative Phosphorylation signaling pathways in aged Spp1‐cKO mice compared to Spp1^fl/fl^ mice (Figure 3F; Figure S5A–C).

Based on the intriguing findings in mice, analysis of a human‐based public database (GSE157827) was conducted. First, major brain cell types were identified using canonical markers (Figure S5D), followed by sub‐clustering of the microglial population (Figure S5E). The SPP1^high^ and SPP1^low^ MG subpopulations were isolated based on SPP1 expression (Figure 3G). Using AUCell, an algorithm to score the activity of regulons in cells (Aibar et al. 2017), the activity of OXPHOS pathways in MG from aged human brains was analyzed. The results showed that the AUC score for OXPHOS in SPP1^high^ MG was significantly higher than that in SPP1^low^ MG (Figure 3H,I). To reinforce this conclusion, we re‐analyzed an independent aging human brain dataset (GSE188545) using AUCell‐based pathway scoring and observed the same pattern: SPP1^high^ microglia exhibit higher oxidative phosphorylation activity than SPP1^low^ microglia (Figure S5F,G). Taken together, these findings suggest that deficiency of Spp1 compromised MG phagocytic function via OXPHOS.

### 2.4 Deficiency of Spp1 Disrupted Microglia Mitochondrial Complex I‐ Dependent Oxidative Phosphorylation in Aged Mice

**FIGURE 4:**
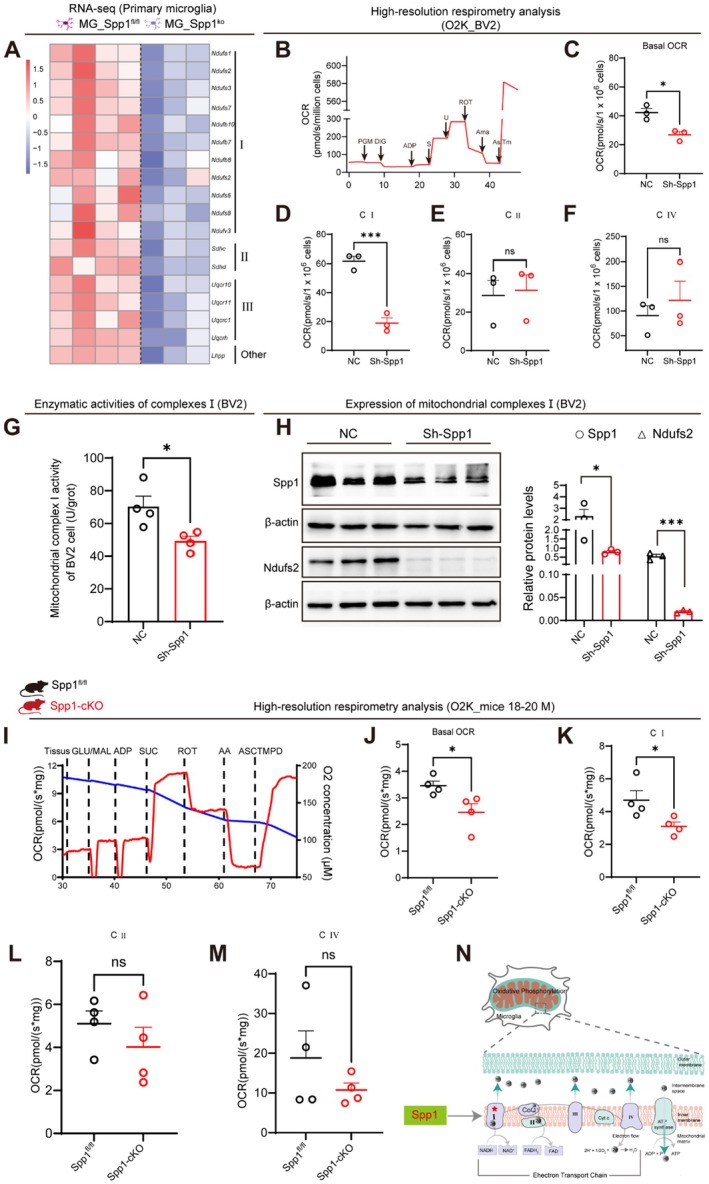
Deficiency of Spp1 in microglia disrupted mitochondrial complex I‐dependent oxidative phosphorylation. (A) Heatmap showing changes in key OXPHOS‐related genes in primary microglia isolated from postnatal day 3 (P3) Spp1^fl/fl^ and Spp1‐cKO mice. (B) Workflow diagram of the Oroboros O2k multi‐dimensional energy metabolism analysis system used to measure OCR in BV2 cell homogenates. (C) Basal OCR in BV2 cells (*n =* 3 per group). (D‐F) Mitochondrial respiration states in BV2 cells: Complex I respiration (D), complex II respiration (E), and complex IV respiration (F) (*n =* 3 per group). (G) Histogram showing the relative mitochondrial complex I enzyme activity (*n =* 4 per group). (H) Western blot analysis showing changes in protein expression levels of Spp1 and Ndufs2 after Spp1 knockdown in BV2 cells (*n =* 3 per group). (I) Workflow diagram of the Oroboros O2k multi‐dimensional energy metabolism analysis system used to measure OCR in hippocampal tissue from Spp1^fl/fl^ and Spp1‐cKO mice. (J) Basal OCR in hippocampal tissue from Spp1^fl/fl^ and Spp1‐cKO mice (*n =* 4 per group). (K–M) Mitochondrial respiration states in hippocampal tissue from Spp1^fl/fl^ and Spp1‐cKO mice: Complex I respiration (K), complex II respiration (L), and complex IV respiration (M) (*n =* 4 per group). (N) Schematic diagram showing Spp1 deficiency disrupting OXPHOS via compromised complex I activity. Data are presented as the mean ± standard error of the mean (SEM). Data were analyzed by unpaired two‐tailed *t*‐tests. *p < 0.05, **p < 0.01, ***p < 0.001, ns = no significant difference. Mouse patterns and experimental apparatus were created using BioRender.

Given that cellular OXPHOS occurs in mitochondria, where complexes (I‐IV) and ATP synthase (complex V) dominate electron transport and ATP generation (Zhao and Xu 2022), transcriptome analysis was performed on MG isolated from Spp1‐cKO and Spp1^fl/fl^ mice to identify the genes involved in OXPHOS. It was shown that the expression of OXPHOS related genes was significantly reduced in Spp1‐deficient MG, especially those encoding subunits of mitochondrial complex I (Figure 4A). Subsequently, the complexes I, II, and IV‐mediated respiratory capacity of Spp1‐deficient BV2 cells was assessed by high‐resolution respirometry (Oroboros Oxygraph‐O2K), with sequential administration of reagents (Figure 4B). Compared to the control, Sh‐Spp1 BV2 cells exhibited decreased basal OCR (Figure 4C). Moreover, the reduction in OCR was dependent on complex I (Figure 4D), but not complex II or IV (Figure 4E,F). Additionally, mitochondrial complex I enzyme activity was decreased in Sh‐Spp1 BV2 cells compared to control cells (Figure 4G). In contrast, the enzymatic activities of complexes II, III, IV, and V remained unchanged (Figure S6A), indicating a selective impairment of complex I. The expression of Ndufs2, a subunit of mitochondrial complex I, was also decreased in Sh‐Spp1 BV2 cells (Figure 4H). Consistent with these respiratory defects, JC‐1 staining analysis revealed a significant reduction in mitochondrial membrane potential in Spp1‐deficient BV2 cells (Figure S6B). These results suggest that the deficiency of Spp1 in MG impairs complex I‐dependent OXPHOS.

To validate the above observations in vivo, a series of analyses were conducted on hippocampal tissue from aged Spp1^fl/fl^ and aged Spp1‐cKO mice (Figure 4I). Basal OCR in the hippocampus of 18‐month‐old Spp1‐cKO mice was reduced compared to that of Spp1^fl/fl^ mice (Figure 4J). Further analysis revealed that the reduction in OCR was dependent on complex I (Figure 4K), but not on complexes II or IV (Figure 4L,M). Importantly, this metabolic impairment appears to be age‐dependent, as young (5‐month‐old) Spp1‐cKO mice showed no significant difference in Complex I‐linked respiration compared to Spp1^fl/fl^ mice (Figure S6C). It is well documented that compromised mitochondrial complex I activity leads to the generation of reactive oxygen species (ROS) (Genova and Lenaz 2015). Therefore, ROS levels were examined in BV2 cells with Spp1 knockdown (LV‐shSpp1), primary cultured MG, and hippocampal tissue from Spp1‐cKO mice using a commercial ROS detection assay kit (Figure S6D). The results showed that Spp1 deficiency led to an increase in ROS levels in BV2 cells, primary MG, and hippocampal tissue of Spp1‐cKO mice, respectively (Figure S6D). Importantly, Pearson's correlation analysis revealed a significant positive correlation between mitochondrial respiration and behavioral performance in both Y‐maze and Morris water maze tests (Figure S6E,F), directly linking the observed metabolic deficits to age‐related memory decline. These findings suggest that the deficiency of MG‐specific Spp1 induces mitochondrial complex I dysfunction, resulting in inhibition of OXPHOS (Figure 4N).

### 2.5 Spp1 Deficiency Impaired AKT Mediated OXPHOS in Microglia and AKT Agonist SC79 Restored Age‐Related Phenotypes of Aged Spp1‐cKO Mice

**FIGURE 5:**
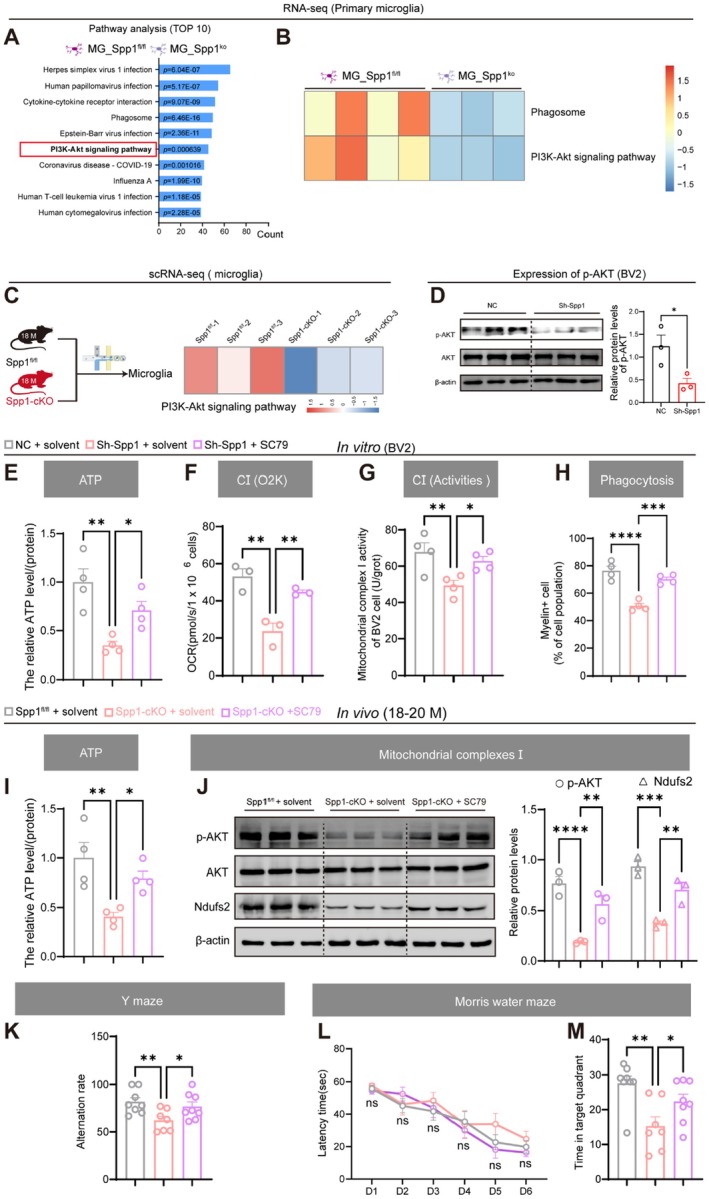
Spp1 deficiency inhibited AKT signaling pathway and administration of AKT agonist reversed manifestation. (A) KEGG pathway analysis of differentially expressed genes from sequencing data of primary microglia isolated from postnatal day 3 (P3) Spp1^fl/fl^ and Spp1‐cKO mice. (B) Heatmap showing GSVA enrichment scores for the “PI3K‐Akt signaling” pathway in primary microglia isolated from postnatal day 3 (P3) Spp1^fl/fl^ and Spp1‐cKO mice. Each column represents one RNA‐seq sample (red indicating up‐regulation, and blue indicating down‐regulation). (C) scRNA‐seq analysis showing the downregulation of PI3K–Akt signaling pathway in the microglial cluster of 18‐month‐old Spp1‐cKO mice compared to controls. (D) Western blot analysis showing the changes in protein expression levels of p‐AKT, AKT after Spp1 knockdown in BV2 cells (*n =* 3 per group). (E) ATP levels (*n =* 4 per group). (F) Mitochondrial complex I‐dependent oxygen consumption rate (OCR) (*n =* 3 per group). (G) Histogram showing the relative mitochondrial complex I enzyme activity (*n =* 4 per group). (H) %Myelin^+^cell (of cell population) = number of phagocytic cells/total cell count (*n =* 4 per group). (I) ATP levels in the hippocampus of Spp1^fl/fl^ + solvent, Spp1‐cKO + solvent, and Spp1‐cKO + SC79 mice (*n =* 3 per group). (J) Western blot analysis showing the changes in protein expression levels of p‐AKT, AKT, and Ndufs2 (*n =* 3 per group). (K) Percentage of correct alternations in the Y‐maze test. (L) Learning curves during MWM training, measured by latency to find the platform. (M) Time spent in the target quadrant during the probe phase of the MWM. Data are presented as the mean ± standard error of the mean (SEM). Two‐way repeated measures ANOVA was used to compare the latency to find the platform in the learning curves (L). Figure D was analyzed using two‐tailed *t*‐tests. Other data were analyzed using one‐way ANOVA and LSD post hoc test. *p < 0.05, **p < 0.01, ns = no significant difference. Mouse pattern was created using BioRender.

After observing that Spp1 deficiency disrupted OXPHOS, the signaling pathway through which Spp1 regulates MG OXPHOS was further investigated. KEGG pathway analysis of DEGs from primary microglia isolated from Spp1^fl/fl^ and Spp1‐cKO mice revealed that the PI3K‐Akt signaling pathway was one of the most enriched pathways (Figure 5A). As shown in Figure 5B, the “PI3K‐Akt signaling” pathway was significantly downregulated in Spp1‐deficient microglia compared to controls. Consistently, scRNA‐seq of 18‐month‐old Spp1‐cKO mice confirmed this signaling defect specifically within the aged microglial cluster (Figure 5C). Consistent with reports that the AKT pathway is a pivotal regulator of mitochondrial function and energy metabolism (He et al. 2022; Yang et al. 2013), we further verified the activation status of AKT at the protein level. Western blot analysis showed that p‐AKT, the active form of AKT, was significantly downregulated in BV2 cells with *Spp1* deficiency (Figure 5D).

Given that p‐AKT was downregulated by Spp1 deficiency, we wondered if activation of AKT by its agonist could rescue the phenotypes manifested in Spp1 KO in vitro and in vivo. Upon treatment with the AKT agonist (SC79), ATP production (Figure 5E), mitochondrial complex I‐dependent OCR (Figure 5F), mitochondrial complex I enzymatic activity (Figure 5G), and phagocytic function (Figure 5H) were restored in Spp1‐deficient BV2 cells. In Spp1‐cKO mice, intraperitoneal administration of SC79 significantly elevated ATP levels in hippocampal tissue compared with those of controls (Figure 5I). Moreover, SC79 treatment elevated the expression of Ndufs2, a key mediator of OXPHOS (Figure 5J). The rescue effect of SC79 treatment on memory deficits in Spp1‐cKO mice was subsequently validated. SC79‐treated mice exhibited a higher correct‐entry rate in the Y‐maze test compared to control mice (Figure 5K). While SC79 treatment did not improve learning performance in the training phase (Figure 5L), SC79‐treated mice spent more time in the target quadrant during the probe phase of the water maze test (Figure 5M). These results indicate that pharmacological activation of the AKT pathway could mitigate age‐related memory deficits induced by Spp1 deficiency by restoring energy metabolism.

### 2.6 Microglia‐Specific Overexpression of Spp1 Rescued the Phenotypes Induced by Spp1 Deficiency Both In Vitro and In Vivo

**FIGURE 6:**
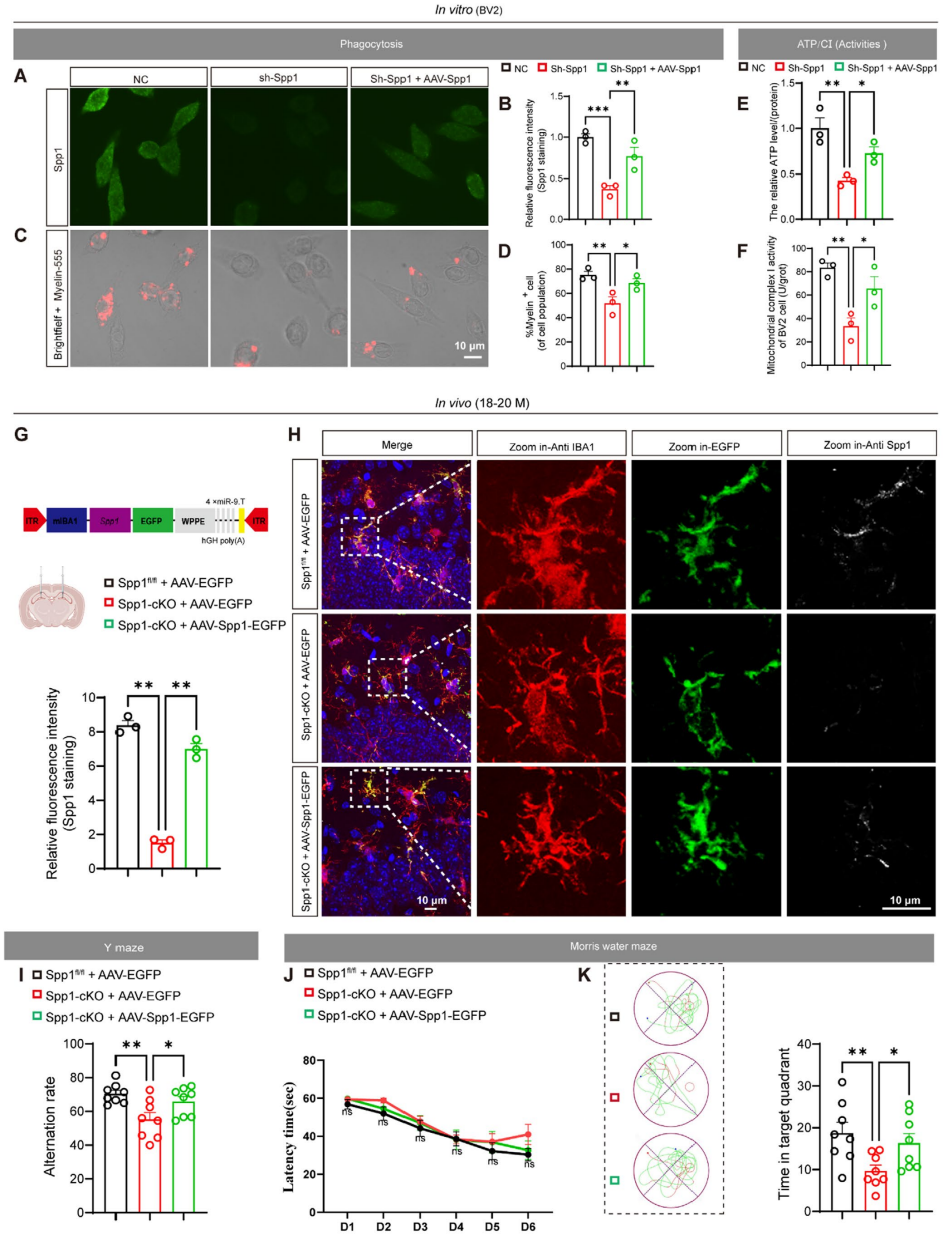
Microglia‐specific Spp1 overexpression restores ATP production, phagocytosis, and memory in Spp1‐deficient models. (A) Representative confocal images of Spp1 immunofluorescence in BV2 cells: Control (NC), Spp1 knockdown (sh‐Spp1), and sh‐Spp1 rescued by AAV‐Spp1. Scale bars = 10 μm. (B) Quantification of relative Spp1 fluorescence intensity (*n =* 3 per group). (C) Representative bright‐field/fluorescence overlays of myelin‐555 uptake (red) by BV2 cells under the three conditions. Scale bars = 10 μm. (D) %Myelin^+^cell (of cell population) = number of phagocytic cells/total cell count (*n =* 3 per group). (E) ATP levels normalized to total protein in control (NC), Spp1 knockdown (sh‐Spp1), and sh‐Spp1 cells rescued by AAV‐Spp1 (*n =* 3 per group). (F) Mitochondrial complex I activity in control (NC), Spp1 knockdown (sh‐Spp1), and sh‐Spp1 cells rescued by AAV‐Spp1 (*n =* 3 per group). (G) Top: Schematic diagram illustrating the experimental strategy for microglia‐specific Spp1 overexpression. Bottom: Relative fluorescence intensity of Spp1 immunostaining in IBA1^+^ microglia (*n =* 3 mice per group). (H) Representative immunofluorescence images showing the expression of AAV‐driven EGFP in IBA1^+^ microglia in the hippocampus. Scale bars = 10 μm. (I) Y‐maze spontaneous alternation rate (*n =* 8 mice per group). (J) Learning curves during MWM training, measured by latency to find the platform (*n =* 8 mice per group). (K) Representative path plots showing the search patterns during the MWM probe trial. (L) Time spent in the target quadrant during the probe phase of the MWM. Data are presented as the mean ± standard error of the mean (SEM). Two‐way repeated measures ANOVA was used to compare the latency to find the platform in the learning curves (J). Other data were analyzed using one‐way ANOVA and LSD post hoc test. *p < 0.05, **p < 0.01, ***p < 0.001. Brain pattern was created using BioRender.

Given the pivotal role of Spp1 in microglial metabolism and cognitive function, we next tested whether its targeted re‐expression could reverse both cellular and behavioral deficits arising from Spp1 deficiency. Compared to the NC group, knockdown of Spp1 led to a dramatic reduction in fluorescence intensity, which was partially restored by AAV‐mediated Spp1 overexpression (Figure 6A,B), confirming that exogenous Spp1 delivered via AAV effectively reinstated BV2 cell Spp1 protein levels. Functionally, Spp1 overexpression partially rescued the phagocytic impairment of BV2 cells induced by Spp1 deficiency (Figure 6C,D). In parallel, Spp1 overexpression partially rescued the decrease in ATP levels and mitochondrial complex I activity in BV2 cells caused by Spp1 deficiency (Figure 6E,F).

Next, we asked whether MG‐Spp1 overexpression could ameliorate the memory deficits observed in Spp1‐cKO mice. MG‐specific overexpression of Spp1 was achieved by stereotaxically injecting rAAV‐mIBA1‐mSpp1‐P2A‐EGFP into the bilateral hippocampi of Spp1‐cKO mice. Three weeks post‐injection, AAV‐driven Spp1 overexpression in Spp1‐cKO mice effectively restored Spp1 expression in MG, with quantification showing levels significantly higher than those in the cKO group (Figure 6G,H). As shown in Figure 6I, Spp1‐cKO mice treated with AAV‐Spp1‐EGFP displayed a significantly higher rate of correct arm entries in the Y‐maze compared to Spp1‐cKO controls, indicating a partial rescue of their memory deficits. In the MWM assay, all groups exhibited similar learning curves (Figure 6J), but in the probe trial, AAV‐Spp1‐EGFP–treated Spp1‐cKO mice spent significantly more time in the target quadrant than Spp1‐cKO controls (Figure 6K), demonstrating a robust recovery of spatial memory.

Together, these data demonstrate that MG‐specific overexpression of Spp1 is sufficient to rescue both the cellular (phagocytosis and ATP production) and behavioral (memory) phenotypes induced by Spp1 deficiency.

## 3 Discussion

In the present study, we found that a population of Spp1‐positive MG was present in the aged mouse as well as human brain. Spp1‐positive MG exhibited enhanced phagocytic capacity. Notably, deficiency of Spp1 in MG specifically exacerbated age‐related memory deficits, without affecting cognitive function in young mice. Mechanistically, for the first time, we demonstrated that during aging, Spp1 maintained microglial phagocytic function by regulating the AKT/mitochondrial complex I pathway. MG‐specific overexpression of Spp1 partially rescues the Spp1‐deficiency‐driven impairments in cellular function (phagocytosis and ATP production) and memory. This study highlighted the crucial role of MG Spp1 in preserving cognitive health and it could serve as the target for combating age‐related memory decline.

MG play a central role in brain aging, serving not only as immune sentinels but also as key modulators of neural homeostasis. Spp1 is a multifunctional protein highly expressed in the CNS, yet its specific role under physiological aging conditions has remained unexplored (Yim et al. 2022). Here, we identified an Spp1‐positive MG subpopulation in aged mouse and human brains. This observation is consistent with scRNA‐seq findings reported by Silvin et al. (2022). Crucially, this conservation extends to metabolic regulation: analysis of independent human datasets revealed that SPP1 expression strongly correlates with OXPHOS activity, mirroring the complex I‐dependent mechanism defined in our mouse model. Microglial Spp1 deficiency exacerbated age‐related memory deficits, whereas its overexpression reversed these cognitive impairments. Previous studies on Spp1 have mainly focused on its role in brain development (Lawrence et al. 2024), ischemic stroke (Spitzer et al. 2022), and AD (Zhou et al. 2020). Our findings, for the first time, provide solid evidence showing that MG‐derived Spp1 plays a pivotal role in brain aging. Several studies have highlighted the dual role of Spp1 in the brain. On one hand, a large‐scale proteomic analysis in AD patients revealed its expression in protective, anti‐inflammatory MG subpopulations (Johnson et al. 2020). Knockout of Spp1 in APP/PS1 mice exacerbated Aβ plaque deposition, suggesting its beneficial role in AD (Rentsendorj et al. 2018). Spp1 has also been shown to exert a protective role in an ischemic stroke mouse model (Ladwig et al. 2019). On the other hand, neutralizing Spp1 in 5 × FAD mice reduced Aβ plaque deposition (Qiu et al. 2023), and increased SPP1 expression in MG was associated with faster cognitive decline in AD patients (Lopes et al. 2024). Additionally, an anti‐Spp1 antibody alleviated blood–brain barrier damage in an acute ischemic stroke mouse model (Spitzer et al. 2022). These studies suggest that Spp1 is a multifunctional protein that may exhibit different functional characteristics depending on disease progression, the types of cells expressing it, and the microenvironment, with our research emphasizing its role in brain aging.

It is well established that effective microglial clearance of neurotoxic debris—such as Aβ aggregates and myelin debris—is essential to maintain neural homeostasis during brain aging (Rim et al. 2024). Although receptors such as TREM2 and complement component C1q have been implicated in microglial clearance of neurotoxic debris, only CD22 has thus far been rigorously validated in aged animals as a regulator of microglial phagocytic function (Pluvinage et al. 2019; Rachmian et al. 2024; Scott‐Hewitt et al. 2024). Notably, targeting CD22—a negative regulator of microglial phagocytosis—in the aged brain restores microglial homeostasis and reverses cognitive decline (Pluvinage et al. 2019). Given our limited knowledge of regulators of microglial phagocytosis in the aging brain, we demonstrated that Spp1 positively regulated microglial phagocytic function. Importantly, the accumulation of brain waste such as Aβ and myelin debris increases, which further disrupts brain homeostasis (Marschallinger et al. 2020; Rodrigue et al. 2009). MG play a crucial role in clearing brain waste, and a reduction in the efficiency of MG in this process further exacerbates brain aging (Pluvinage et al. 2019). Grajchen et al. (2020) suggested that the accumulation of myelin debris in the brain could trigger an inflammatory response. In line with this, the enhanced inflammatory response observed in the brains of aged Spp1‐cKO mice indicates that the reduction in myelin debris phagocytosis due to Spp1 deficiency in MG may contribute to increased neuroinflammation. Our findings reveal that Spp1 modulates MG phagocytic function to influence the clearance of waste (e.g., myelin debris) in the brain, thereby mediating the process of brain aging.

Microglial phagocytosis is an energy‐dependent process that depends on both glycolysis and OXPHOS to meet its ATP demands (Kim et al. 2024). However, the upstream regulators that coordinate these metabolic pathways of MG within the aged brain remain poorly understood. In the present study, we found that MG with Spp1 deficiency exhibited decreased ATP levels, suggesting that Spp1 plays a crucial role in regulating cellular energy metabolism, thereby affecting its phagocytic function. Using RNA‐seq and Seahorse energy metabolism analyzer, we further revealed that the energy decline induced by Spp1 KO of MG was driven by impaired OXPHOS, but not glycolysis. Supporting our observation, studies have indicated that in the context of aging and neurodegenerative diseases, OXPHOS dysfunction is a primary cause of energy insufficiency in MG (Bennett and Liddelow 2019), suggesting that Spp1 plays an essential role in maintaining OXPHOS‐mediated energy metabolism in MG.

OXPHOS generates ATP through the coordinated action of the electron transport chain (ETC), which consists of large multi‐subunit complexes I–IV and ATP synthase located on the inner mitochondrial membrane (Zhao and Xu 2022). Complex I initiates electron transfer from NADH to the ETC and is essential for maintaining mitochondrial respiration and ATP synthesis (Zhao and Xu 2022). Our RNA‐seq data revealed that Spp1‐deficient MG exhibited significant downregulation of genes encoding complex I subunits, accompanied by a marked decrease in complex I–linked OCR in O2K assays. This finding implicates Spp1 as an upstream regulator whose absence disrupts complex I activity and compromises ATP generation. Multiple lines of evidence from diverse models corroborate the centrality of complex I in microglial bioenergetics. Mora‐Romero et al. (2024) reported that MG from complex I‐deficient mouse models exhibited metabolic dysfunction. Moreover, in AD and PD patients, inhibition of complex I activity and reduced OXPHOS‐mediated ATP production have been observed (Franco‐Iborra et al. 2018; Hroudova et al. 2014). Moreover, a recent study showed that aberrant activity of complex I in MG from multiple sclerosis mice triggered ROS release and neuropathological damage (Genova and Lenaz 2015). In parallel, we observed elevated ROS production in Spp1‐deficient microglia, consistent with electron leakage due to dysfunctional complex I. These findings collectively highlight the pivotal role of complex I in regulating MG function. However, the specific upstream factors that influence complex I expression and activity remain unclear. Based on our results, our study is the first to reveal that Spp1 is a key upstream regulator of complex I activity in MG.

Among the upstream factors influencing the expression and activity of complex I, the AKT signaling pathway is a well‐known axis that regulates mitochondrial function, especially OXPHOS (He et al. 2022). In the present study, RNA‐seq analysis revealed that the AKT signaling pathway was altered by MG Spp1 deficiency. The active form of p‐AKT was down‐regulated by Spp1 deficiency in BV2 cells. Other studies have demonstrated that Spp1 is an important upstream activator of the AKT signaling pathway, and Spp1 knockout suppresses AKT signaling (Bai et al. 2023; Eun et al. 2023), which is consistent with our findings. Based on our results and previous studies, we propose that MG Spp1 knockout leads to down‐regulation of the AKT signaling pathway, which in turn inhibits the expression of genes related to mitochondrial complex I, ultimately decreasing OXPHOS, ATP production, and MG function. The identified pathway through which MG Spp1 knockout impairs AKT signaling and OXPHOS paves the way for pharmacological rescue of the phenotypes observed in Spp1‐deficient mice. We administered SC79, a p‐AKT agonist, to Spp1‐cKO mice and found that SC79 restored OXPHOS, MG phagocytic capacity, and rescued the memory deficits in the mice. Uniquely, distinct from established aging drivers like the NAD^+^/sirtuin axis or TREM2–APOE pathways (Liu et al. 2025), our work identifies Spp1 as a specific metabolic “checkpoint” that operates in parallel with these canonical mechanisms to sustain Complex I‐dependent respiration. These findings suggest that modulating Spp1‐mediated energy metabolism could serve as a novel therapeutic strategy for combating age‐related memory decline.

Notably, there are still several gaps that need to be addressed to expand the application of the current study. First, our experimental design did not differentiate between intracellular and secreted Spp1 isoforms. Thus, future work is needed to dissect their specific contributions and the potential role of sSpp1‐mediated integrin/CD44 signaling. Second, our findings demonstrate that microglial‐specific Spp1 deletion leads to memory impairment, implying a functional breakdown in microglia–neuron crosstalk. However, the specific neuronal deficits (e.g., synaptic loss, reduced trophic support, or secondary neurotoxicity) resulting from microglial metabolic failure remain to be characterized. Third, developing feasible approaches to selectively elevate MG Spp1 expression is needed. Finally, to bridge the gap between rodent models and human pathology, validating these metabolic signatures in human‐derived platforms, such as brain organoids, is crucial. Furthermore, identifying the specific upstream molecular cues that trigger Spp1 induction in aging microglia will provide a more complete understanding of this regulatory circuit.

## 4 Materials and Methods

### 4.1 Animals

The *Cx3cr1*‐CreERT2 (Stock No. C001247) and *Spp1*‐flox (Spp1^fl/fl^) (Stock No. S‐CKO‐05240) mice were obtained from Cyagen Biosciences Co. Ltd. (China). The Spp1^fl/fl^ mice were generated with loxP sites flanking exons 5–8 of the Spp1 gene. The Spp1^fl/fl^ mice were crossed with Cx3cr1‐CreERT2/+ mice to generate *Cx3cr1*‐CreERT2/+::Spp1^fl/fl^ animals. C57BL/6J mice (WT) were purchased from the Animal Center of Shanxi Medical University (China). All animals were housed in a specific pathogen free environment with a 12:12 h light/dark cycle, temperature maintained at 22°C ± 2°C, and humidity at 50% ± 10%, with ad libitum access to water and food. The animal procedures were approved by the Medical Ethics Committee of Shanxi Medical University (Ethical code: SYDL2024033), and all procedures followed the guidelines established by the welfare ethics committee for laboratory animals. Animals were randomly assigned to different groups using the random number generator function (RANDBETWEEN) in Microsoft Excel. Researchers were blinded to group assignments. Following the criteria of Sato‐Hashimoto et al. (2019), 16–24‐month‐old mice were designated as aged.

### 4.2 Human Brains

Appropriate consent was secured prior to the collection of all human brain tissue samples. Human brains were collected from the Human Brain Tissue Bank at Shanxi Medical University. Research protocol involving human brain samples was reviewed and approved by the Ethics Committee of Shanxi Medical University (Ethical code: 2023ptll001). The donor‐level demographics and postmortem details (age, sex, clinical diagnosis, postmortem interval, and regions of interest) are provided in Table S3.

### 4.3 Tamoxifen Administration

Two distinct tamoxifen induction protocols were employed depending on the experimental design. For in vivo studies, Cre recombination was induced in young adults to avoid developmental confounds, following the method described by Wang et al. (2022). Briefly, mice at 1 month of age were fed tamoxifen‐containing chow (Cat. No. TAM500, SYSE) for 4 weeks, followed by a switch to regular chow. For in vitro experiments using primary microglia, neonatal induction was performed as previously described (Hu et al. 2022). Tamoxifen (Cat. No. T5648, Sigma Aldrich; 100 μg) dissolved in corn oil (Cat. No. F2214830, Aladdin) was administered via intraperitoneal injection on postnatal days 1 and 2 (P1 and P2) prior to cell isolation.

### 4.4 Microglia Primary Cultures

Primary MG were isolated as described previously (Hu et al. 2022), with slight modifications. Briefly, brain tissues were digested with papain (Worthington) at 37°C for 20 min. Dissociated cells were cultured in DMEM supplemented with 10% FBS and 1% P/S on poly‐D‐lysine (PDL)‐coated flasks. After ~11 days, microglia were harvested by shaking (240 rpm, 37°C, 1.5–2 h) and replated for experiments. Detailed information regarding reagents is provided in Table S2.

### 4.5 Measurements of ATP

BV2 cell, hippocampi, and primary MG from Spp1^fl/fl^ and Spp1‐cKO mice were homogenized. ATP levels were then quantified using an ATP bioluminescence assay kit (Cat. No. S0027, Beyotime Biotechnology). ATP measurements were normalized to protein content for comparison.

### 4.6 Metabolic Extracellular Flux Analysis

The XF Seahorse extracellular flux analyzer (Seahorse Bioscience, Billerica, MA, USA) was employed to monitor real‐time alterations in ECAR and OCR of primary MG, as described in previous studies (Hu et al. 2022). In brief, primary MG were incubated with 2‐DG (5 mM) or 3‐BrPA (50 μM) for 3 h. Assays were performed using the XF Cell Mito Stress Kit (Cat. No. #1030100‐100) and Glycolytic Rate Assay Kit (Cat. No. #103017‐100) in specific running buffers supplemented with glucose, pyruvate, and glutamine as required. Data were normalized to cell counts determined by DAPI staining.

### 4.7 CD11b Magnetic Labeling and Separation From Brain Tissue

As described in the research by Ocañas SR (Ocanas et al. 2022), MG were isolated from the brains of Spp1^fl/fl^ or Spp1‐cKO mice. Brain tissues were enzymatically digested using the Neural Tissue Dissociation Kit (Miltenyi Biotec) on a gentleMACS Octo dissociator (37°C, 21 min). The resulting single‐cell suspensions were filtered, labeled with CD11b MicroBeads (Miltenyi Biotec), and passed through MS columns to enrich for CD11b^+^ microglia. Detailed information regarding reagents is provided in Table S2.

### 4.8 RNA‐Seq and Data Analysis

The sorted MG were quickly frozen in liquid nitrogen and sent to BGI (Shenzhen, China) for transcriptome analysis. Briefly, poly(A)‐enriched mRNA was converted into single‐stranded circular DNA libraries for sequencing. After constructing the library, quality control was performed to ensure it met sequencing standards, followed by sequencing analysis. Differential genes were identified in R using a threshold of *p* < 0.05 and |log_2_FC| > 0.25. Gene Set Enrichment Analysis (GSEA) of these differential genes was performed using the gseGO and gseKEGG functions from the clusterProfiler package (v4.7.1.003). Visualization of the results was performed using the gseaplot2 function from the enrichplot package (v1.18.4).

### 4.9 In Vitro Phagocytosis Assay

It was assessed following the method described by Pluvinage et al. (2019). Primary microglia were isolated and cultured from Spp1^fl/fl^ and Spp1‐cKO mice. AlexaFluor555‐labeled myelin (myelin‐555) (5 μg/mL) was added to the culture medium and incubated for 3 h. After incubation, MG were fixed with 4% PFA and subjected to immunofluorescence staining. The positive rate and fluorescence intensity of phagocytosis were analyzed using ImageJ software. The percentage of phagocytic cells (%Phagocytic) was calculated as the ratio of phagocytic cells to the total number of cells. The relative myelin‐555 intensity in phagocytic cells was determined by dividing the total fluorescence intensity by the total number of phagocytic cells.

### 4.10 In Vivo Phagocytosis Assay

Following the method described by Marschallinger et al. (2020), Myelin‐555 (1 μL, 25 mg/mL) was stereotaxically injected into the hippocampus (AP −1.7 mm, ML +0.7 mm, DV −2.04 mm) at a rate of 200 nL/min. After 48 h, mice were perfused with 4% PFA. Coronal brain sections (40 μm) were immunostained for Iba1 and Spp1 to quantify myelin uptake in Spp1^+^/Iba1^+^ and Spp1^−^/Iba1^+^. Detailed methodological descriptions are provided in the Supporting Informations and Methods.

### 4.11 3D Reconstruction of Confocal Images

The FV3000 confocal images were imported into IMARIS 9.5 software, where the 3D reconstruction feature was used to create three‐dimensional renderings of the segmented images, and the rendering parameters were adjusted to optimize the visual quality.

### 4.12 RT‐qPCR

RNA was extracted from mouse brain tissue using the TRIzol method. cDNA was synthesized from the RNA using a reverse transcription kit (Cat. No. RR047A, TaKaRa). Quantitative real‐time PCR was performed with SYBR Premix Ex Taq II (Cat. No. RR820A; TaKaRa, Dalian, China). Cycling conditions: initial denaturation 95°C for 30 s, followed by 40 cycles of 95°C for 5 s and 60°C for 30 s; a melt‐curve analysis was included to verify amplicon specificity. Relative expression was calculated by the $2^{-\Delta \Delta C_{t}}$ method with β‐actin as the reference gene. The primer sequences are listed in Table S4. The primers were synthesized by Huada Gene Company (Beijing, China).

### 4.13 Western Blot

Proteins were extracted from tissues or cells in ice‐cold RIPA buffer (Cat. No. HY‐K1001, MCE) with protease (Cat. No. HY‐K0010, MCE) and phosphatase (Cat. No. HY‐K0023, MCE) inhibitors (cleared by centrifugation [12,000 *g*, 15 min, 4°C]), and their concentrations were determined using the BCA method (Cat. No. cw0014, CWBIO). Equal amounts of protein (20–30 μg per lane) were separated by SDS‐PAGE and transferred onto PVDF membranes (Cat. No. IPVH00010, Millipore). The membranes were blocked with 5% non‐fat milk in TBST (or 5% BSA for phospho‐blots) and incubated overnight at 4°C with primary antibodies. After washing, the membranes were incubated with HRP‐conjugated secondary antibodies for 1 h at room temperature. Protein bands were visualized using an ECL detection system and imaged with a multi‐functional imaging system (Bio‐Rad, Shenhuabio). The grayscale values of the target and reference genes were analyzed using ImageJ. Details of the antibodies are provided in Table S1.

### 4.14 Enzyme‐Linked Immunosorbent Assay

TNF‐α, IL‐6, and IL‐1β levels in hippocampal tissue from Spp1^fl/fl^ or Spp1‐cKO mice were measured using ELISA kits for TNFα (Cat. No. EM0183, Jining Bio), IL‐6 (Cat. No. EM0121, Jining Bio), and IL‐1β (Cat. No. EM0109, Jining Bio), according to the manufacturer's instructions.

### 4.15 Stereotaxic Adeno‐Associated Virus (AAV) Injection

Spp1‐overexpressing AAV (rAAV‐mIBA1‐mSpp1‐P2A‐EGFP‐WPRE‐4 × miR9T, AAV/11, titer: ≥ 1.00 × 10^13^ vg/mL) and control AAV (rAAV‐mIBA1‐P2A‐EGFP‐WPRE‐4 × miR9T, AAV/11, titer: ≥ 1.00 × 10^13^ vg/mL) were obtained from Brain Case (Shenzhen, China). Following published protocols (Serrano et al. 2024), viruses were stereotaxically injected into the mouse hippocampus. Briefly, mice were anesthetized with isoflurane (4% for induction, 1.5%–2% for maintenance in oxygen) and secured in a stereotaxic frame. A total of 0.5 μL of virus was delivered at a rate of 0.03 μL/min to coordinates relative to bregma: AP –1.7 mm, ML +0.7 mm, and DV –2.04 mm. After injection, the needle was left in place for 15 min before being withdrawn slowly. Mice were allowed to recover on a heating pad and were sacrificed 3 weeks later by transcardial perfusion with PBS followed by 4% paraformaldehyde, after which brains were collected for analysis.

### 4.16 Public Sequencing Data Analysis

Public data were downloaded from the GEO database (http://www.ncbi.nlm.nih.gov/geo/); detailed information can be found in the Supporting Informations and Methods.

### 4.17 Y‐Maze Test

Mice were placed in a Y‐shaped maze with three arms, each 35 cm in length, and allowed to explore freely for 5 min. An arm entry was defined as placement of all four paws fully within an arm (partial entries were not counted) (Mandai et al. 2020). The number of entries into each arm and the sequence of arm entries were recorded. Spontaneous alternation, defined as consecutive entries into all three arms, was calculated as an indicator of spatial working memory. Spontaneous alternation (%) was calculated as the number of triads of three consecutive entries into three different arms divided by (total arm entries − 2) × 100 and was used as an index of spatial working memory. Cages were coded, and experimenters performing handling, testing, and scoring were blinded to genotype and treatment until data analysis was completed.

### 4.18 Morris Water Maze

Mice were trained to locate a hidden platform submerged in a circular pool (120 cm in diameter) filled with opaque water. Training was conducted over 5–6 days, with four trials per day. Each trial lasted up to 60 s, during which the time taken to find the platform (escape latency) was recorded. A probe trial was conducted 24 h after the final training session, during which the platform was removed, and the time spent in the target quadrant was measured. To ensure objectivity, all behavioral procedures and data analyses were performed in a double‐blind manner, with investigators unaware of the specific genotypes or treatments of the mice.

### 4.19 Quantification and Statistical Analysis

The experimental data in this study are presented as means ± standard error of the mean (SEM) and were statistically analyzed using GraphPad Prism 9.0. For comparisons between two groups, an independent samples *t*‐test was used. For comparisons among more than two groups, one‐way ANOVA was performed, followed by LSD post hoc tests. Data were assessed for normal distribution and homogeneity of variance, and a *p*‐value of less than 0.05 was considered statistically significant. Pathway activity comparisons between the SPP1^high^ and SPP1^low^ MG subpopulations were conducted using the Wilcoxon rank‐sum test in R. The learning curves from the Morris water maze were analyzed using repeated measures ANOVA.

Other methods are shown in Supporting Informations and Methods.

## 5 Conclusion

Summarily, in the present study, we found that Spp1 is a critical mediator of microglial phagocytosis via AKT/mitochondrial complex I–dependent OXPHOS, and that modulating Spp1 expression can reverse memory deficits associated with brain aging.

## Author Contributions

Conception and design of the study: M.W., A.H., X.G., H.W., A.L., and L.L. Writing and editing the manuscript: M.W., X.G., and C.Z. Sequencing data download and analysis: A.H. Performing the experiments: M.W., A.H., J.Y., and Y.C. Drawing summary plot: M.W. and Y.C. Statistical analysis: M.W., A.H., and Y.C. Obtaining funding, administrative, and supervision: C.Z., L.L., and K.‐L.L. The final manuscript has been read and approved by all authors.

## Funding

This study was supported by grants from the Natural Science Foundation of China (Nos. 82301759, 82571379), the Natural Science Foundation of Shanxi Province (Nos. 20210302123299, 20210302123291, 20210302124561, 202203021211238), the International Science and Technology Cooperation Program of Shanxi Province (No. 202104041101026) and the Shanxi Scholarship Council of China (No. 2024–079), and the Shanxi Province Higher Education “Billion Project” Science and Technology Guidance Project.

## Ethics Statement

The animal procedures were approved by the Medical Ethics Committee of Shanxi Medical University (Ethical code: SYDL2024033), and all procedures followed the guidelines established by the welfare ethics committee for laboratory animals. Research protocol involving human brain samples was reviewed and approved by the Ethics Committee of Shanxi Medical University (Ethical code: 2023ptll001).

## Conflicts of Interest

The authors declare no conflicts of interest.

## Supporting information


**Figure S1:** Spp1 expression is significantly upregulated in the aged brain and microglia.
**Figure S2:** Microglia‐specific Spp1 deletion reduces Spp1 signal but not hippocampal microglia density.
**Figure S3:** Spp1 knockdown impaired microglial phagocytosis.
**Figure S4:** Characterization of cell viability and metabolic pathways in *Spp1*‐deficient primary microglia.
**Figure S5:** Single‐cell transcriptomic analysis of aged Spp1‐cKO mice and vliadation in human aging datasets.
**Figure S6:** Spp1 deficiency induces specific mitochondrial Complex I dysfunction and correlates with cognitive decline.
**Table S1:** Key Resources Table of Antibodies.
**Table S2:** Key Resources Table of Reagents.
**Table S3:** Donor characteristics and postmortem details of human hippocampal samples.
**Table S4:** RT–qPCR primer sequences.

## Data Availability

The raw RNA‐seq data generated in this study have been deposited in the Genome Sequence Archive (GSA) at the National Genomics Data Center, China National Center for Bioinformation/Beijing Institute of Genomics, Chinese Academy of Sciences (GSA: CRA033531) and are publicly accessible at https://ngdc.cncb.ac.cn/gsa. All other data supporting the findings of this study are available from the corresponding author upon reasonable request. *Code availability*: The code is available upon request (contact the corresponding author).
