# Supplementary material for: Deficiency of Microglial‐Derived Spp1 Exacerbates Age‐Related Memory Decline by Impairing Mitochondrial Complex I Function
Source: Aging Cell. 2026 Jan 18;25(2):e70378. doi: 10.1111/acel.70378 (PMC12813269; doi:10.1111/acel.70378)
Supplement: Supplementary file 1 — Figure S1: Spp1 expression is significantly upregulated in the aged brain and microglia. Figure S2: Microglia‐specific Spp1 deletion reduces Spp1 signal but not hippocampal microglia density. Figure S3: Spp1 knockdown impaired microglial phagocytosis. Figure S4: Characterization of cell viability and metabolic pathways in Spp1‐deficient primary microglia. Figure S5: Single‐cell transcriptomic analysis of aged Spp1‐cKO mice and vliadation in human aging datasets. Figure S6: Spp1 deficiency induces specific mitochondrial Complex I dysfunction and correlates with cognitive decline. Table S1: Key Resources Table of Antibodies. Table S2: Key Resources Table of Reagents. Table S3: Donor characteristics and postmortem details of human hippocampal samples. Table S4: RT–qPCR primer sequences. [file ACEL-25-e70378-s001.pdf]

Supporting Information

Deficiency of microglial-derived Spp1 exacerbates age-related memory decline by impairing mitochondrial complex I function

Meiling Wang[1,2], Yumin Chang[1,2], Aojie He[1,2], Jing Yang[1,2], Ang Li[6], Hongqin Wang[3], Kah-Leong Li[7], Xing Guo[4,5], Chengwu Zhang[1,2*], Li Lu[1,2*]*

Supplementary Materials and Methods

Immunohistochemistry

Mice were deeply anesthetized with isoflurane, and their hearts were exposed for perfusion through the left ventricle, first with PBS, followed by 4% paraformaldehyde (PFA), until the liver turned pale and the mouse became rigid. The brains were removed, post-fixed in 4% PFA for 12 hours, and dehydrated in a sucrose gradient (15%, followed by 30%) before being embedded in OCT, flash-frozen in liquid nitrogen, and stored at -80°C. Frozen brains were equilibrated at -20°C for 2 hours before sectioning coronally at a thickness of 40 μm using a cryostat. Sections were collected from the region 1.46 to 2.46 mm posterior to the bregma. Sections were stored in antifreeze buffer at -20°C until use. For immunofluorescence, sections were washed with PBS, permeabilized with 0.3% Triton-X 100 (for cytoplasmic or nuclear proteins), blocked with 5% donkey serum, and incubated overnight at 4°C with primary antibodies (anti-Spp1, anti-GFAP, anti-IBA1, or anti-NeuN). After washing, sections were treated with secondary antibodies (donkey anti-goat 594, donkey anti-mouse 488, donkey anti-rabbit 488, or anti-rabbit Cy5 for IBA1) and mounted with DAPI-containing medium. For human brain tissue sections, Tyramide Signal Amplification (TSA) was used for imaging, according to the manufacturer's instructions (Cat. No. AWI0697, Abiowell). Imaging was performed using a confocal microscope at 40×, 60×, and 100× magnifications, and co-localization of Spp1 with IBA1, NeuN, and GFAP was analyzed using ImageJ software. Details of the antibodies are provided in Supplementary Table S1.

RNA in situ hybridization

RNAScope was performed on brain sections using the Multiplex Fluorescent Reagent Kit v2 (Cat. No. 323100, ACD). Briefly, frozen brain sections (stored at −80°C) were washed with 1× PBS, baked at 60°C for 30 minutes, and then fixed in 10% neutral buffered formalin (NBF) for 15 minutes at 4°C. Following fixation, the brain sections were dehydrated in 50%, 70%, 100%, and 100% ethanol, with each step lasting 5 minutes at room temperature. The sections were then treated with H2O2 for 10 minutes at room temperature, followed by rinsing with distilled water to remove H2O2. After

washing, the sections were incubated with the primary antibody (anti-IBA1) overnight at 4°C. After removing the primary antibody with PBST, the sections were incubated with Protease Plus for 40 minutes at 40°C in a humidified hybridization oven. The brain sections were then hybridized with the Spp1 mRNA probe (RNAscope™ Probe- Mm-Spp1 ACD, Cat. No. 435191-C1) for 2 hours at 40°C in the humidified hybridization oven. Probe amplification was performed according to the manufacturer's instructions using AMP1, AMP2, and AMP3. C1-HRP-labeled probes were visualized using TSA-570 at a 1:1000 dilution, with a 30-minute incubation at 40°C. The secondary antibody (TSA-520) was applied to the sections and incubated at room temperature for 30 minutes. Counterstaining was performed with DAPI for 30 seconds, followed by immediate mounting with Prolong Gold Antifade Mountant (Cat. No. P36930, Thermo Fisher Scientific). Imaging was conducted using a confocal microscope with 40×, 60×, and 100× objectives.

BV2 cell cultures and transfection

BV2 cells, a murine microglial cell line, were cultured in DMEM medium containing 10% FBS and 1% P/S. The Spp1 and control short hairpin RNAs (shRNAs) were purchased from Hanheng Biological Co. (China). To establish Spp1-knockdown BV2 cells, lentiviral infection was used. To overexpress Spp1, an adenoviral vector encoding the mouse Spp1 gene (Gene ID: 20750) was constructed by Shanghai GeneChem Co., Ltd.

Aβ-FITC Uptake Assay

Aβ–FITC uptake assay adapted from Wang et al. (Wang et al., 2022). Primary microglia from $Spp1^{fl/fl}$ and Spp1-cKO littermates were plated with equal numbers of viable cells (5×10^4 cells/well). Cells were incubated with FITC-labeled Aβ(1–42) oligomers (1 μg/mL) for 2 h at 37 °C/5% CO_2, then washed thoroughly with PBS to remove extracellular Aβ. Cells were harvested and analyzed by flow cytometry populations were quantified; the %$A\beta^+$ fraction was calculated as $A\beta^+$ cells / total cells × 100.

Measurement of Inflammatory Cytokines

Culture supernatants from primary microglia were collected and centrifuged (1,000 × g, 10 min). Secreted TNF-α, IL-6, and IL-1β levels were quantified using ELISA kits (EM0183, EM0121, EM0109; Jining Bio) per manufacturer instructions, with absorbance read at 450 nm.

High resolution respirometry

A Oxygraph O2K instrument and DatLab software (Oroboros Instruments, Innsbruck, Austria) were used to measure oxygen consumption rates(Dominguez-Lopez et al., 2023; Mann et al., 2019). (1) *In vitro*: BV2 cells suspensions were centrifuged at 350 × g for 5 minutes. The supernatant was discarded, and the cell pellet was resuspended in 200 μL of MIR05. The resuspended cells were then added to the sample chamber. The

titration protocol was used (See more detailed information below), with reagents added sequentially after stabilization at each step. (2) *In vivo*: The hippocampus from Spp1$^{fl/fl}$ or Spp1-cKO mice were rapidly dissected on ice and weighed. The tissue was minced, homogenized in 1 mL MIR05 using a manual glass homogenizer until a uniform suspension was obtained. For each O2K chamber, 10 mg of tissue homogenate was used. After calibrating the chambers with MIR05, 1 mL of MIR05 was added, followed by 1 mL of tissue homogenate, and the chamber was sealed for analysis. The titration protocol was used (See more detailed information below), with reagents added sequentially after stabilization at each step.

High resolution respirometry of BV2 cell (Titration protocol)

Steps	Reagent	Final concentration	Volume
1 NAD^+-linked substrates	Malate-M	2 mM	10 µl
	Pyruvate-P	5 mM	5 µl
	Glutamate-G	10 mM	10 µl
2 Permeabilization of cell membranes	Digitonin-Dig	5 µg/mL/steps	1 µl/steps
3 Activation of ATP synthase	$MgCl_2$	0.6 mol	10 µl
	ADP-D	2.5 mM	10 µl
4 CII-linked substrate	Succinate-S	10 mM	20 µl
5 maximal-uncoupled respiration	FCCP-U	0.5 µM/steps	1 µl/steps
6 inhibit CI activity	Rotenone-Rot	0.5 µM	1 µl
7 inhibit CIII activity	Antimycin A-Ama	2.5 µM	1 µl
8 CIV-linked substrates	Ascorbate-As	2 mM	5 µl
	TMPD-TM	0.5 mM	5 µl

High resolution respirometry of brain tissue (Titration protocol)

Steps	Reagent	Final concentration	Volume
State 1 NAD^+-linked substrates	Glutamate-G	10 mM	10 µl
	Malate-M	2 mM	10 µl
State 2 complex I respiration	ADP-D	5 mM	20 µl
State 3 test complex II activity	Succinate	10 mM	20 µl
State 4 inhibit complex I activity	Rotenone	1 µM	2 µl
State 5 inhibit complex III activity	Antimycin A-AA	1 µM	0.4 µl
State 6 CIV-linked substrates	Ascorbate	5 mM	12.5 µl
	TMPD	0.5 µM	0.5 µl

Measurement of ROS

(1) *In vitro*: Primary microglia and BV2 cells were seeded in 24-well plates and allowed to reach 80-90% confluence before conducting ROS detection. The DCFH-DA probe (Cat. No. S0033S, Beyotime Biotechnology) was diluted at a 1:1000 ratio and added to each well (500 µL per well), followed by incubation at 37°C for 20 minutes. After incubation, the cells were washed three times with serum-free medium, each wash lasting 5 minutes. The cells were then treated with trypsin for digestion and resuspended, and 10 µL of the cell suspension was taken for cell counting. Finally, a multi-function microplate reader was set with the excitation wavelength at 488 nm and the emission wavelength at 525 nm for detection. The data were normalized to the cell count before analysis. (2) *In vivo*: Hippocampi and cortices were extracted from Spp1$^{fl/fl}$ and Spp1-cKO mice, and homogenized on ice in buffer A at a ratio of 1 mL per 50 mg of tissue. The homogenate was centrifuged at 100 × g for 3 minutes at 4°C, and the supernatant was carefully transferred to another EP tube. A 200 µL aliquot of the supernatant was mixed with 2 µL of the DHE probe (Cat. No. HR882, Baiao Lai Bo) and incubated in

the dark at 37°C for 25 minutes. Simultaneously, the microplate reader was set to the appropriate parameters (excitation wavelength 488-535 nm, emission wavelength 610 nm). After incubation, the samples were immediately analyzed. Protein concentrations were measured using the BCA method, and ROS levels were calculated using the formula: ROS intensity = fluorescence intensity/protein concentration (mg).

BV2 treatment

The method was adapted from Lin SP et al. (Lin et al., 2023). For AKT activation, BV2 cells were pretreated with 5 μM SC79 for 30 minutes.

Enzyme activities of mitochondrial Complex I–V

BV2 cell pellets were collected, and the enzymatic activities of mitochondrial complexes I–V were assessed using specific commercial assay kits (Elabscience, China) according to the manufacturer's instructions. The specific kits used were: mitochondrial complex I activity assay kit (Cat. No. E-BC-K834-M), mitochondrial complex II activity assay kit (Cat. No. E-BC-K835-M), mitochondrial complex III activity assay kit (Cat. No. E-BC-K836-M), mitochondrial complex IV activity assay kit (Cat. No. E-BC-K837-M), and mitochondrial complex V activity assay kit (Cat. No. E-BC-K838-M). All enzyme activity measurements were normalized to the total protein content of the respective samples.

Drug treatment

The method was adapted from Chen C et al. (Chen et al., 2024; Guo et al., 2024). For AKT activation, mice were intraperitoneally injected with SC79, a specific AKT agonist (Glpbio, cat: GC11645).

Analysis of Spp1 Expression in Aged and Young Microglia

Gene expression matrices for aged MG were downloaded from the GEO database (GSE99074 for human and GSE156762 for mouse). Gene names were converted using the clusterProfiler package (V4.7.1.003) in R, along with org.Hs.eg.db (V3.16.0) for human data and org.Mm.eg.db (V3.16.0) for mouse data. For human MG, SPP1 read counts were directly extracted and compared between young and aged groups. For mouse MG, differentially expressed genes were filtered using a threshold of $p < 0.05$ and $|\log2FC| > 0.25$. Volcano plots were generated using the ggplot2 package (V3.4.2).

Enrichment Analysis of SPP1^{high} Microglia in Aged Human Brains

Single-cell RNA sequencing data of MG from aged human brains were downloaded from the GEO database (GSE157827). In R, the Seurat package (V4.4.0) was used for data processing and annotation of MG based on specific marker genes. MG were then classified into two groups based on SPP1 expression levels: SPP1^{high} and SPP1^{low}. GO enrichment analysis was performed to identify the functional characteristics of the SPP1^{high} MG subgroup.

The method for analyzing metabolic pathway activity scores was adapted from the studies by Aibar et al. (Aibar et al., 2017). AUCell (Area Under the Curve Cell scoring)

is a method used to assess gene set activity in single-cell RNA sequencing data. It calculates a score based on the area under the curve for the expression of a specific gene set within each cell, thereby evaluating the activity of that gene set at the single-cell level. Single-cell pathway analysis was performed using AUCell v1.16.0, with metabolic pathways such as OXPHOS downloaded from KEGG (https://www.genome.jp/kegg/pathway.html) used to calculate activity scores, expressed as AUC scores. The AUC score matrix was then imported into Seurat for generating violin plots and tSNE plots.

Single-Cell RNA Sequencing (scRNA-seq)

Hippocampal tissue from 18-month-old Spp1$^{fl/fl}$ and Spp1-cKO mice was enzymatically and mechanically dissociated to generate a heterogeneous single-cell suspension. Viable cells were immediately processed using the 10x Genomics Chromium platform and sequenced on an Illumina system. Raw data were processed using the Cell Ranger pipeline and subsequently analyzed using the Seurat package. Following cell annotation, the microglia cluster was isolated for GSVA, which identified differential activity in the Oxidative Phosphorylation and PI3K-Akt signaling pathways between the Spp1-cKO and control microglia.

In vivo phagocytosis assay

Following the method described by Marschallinger J et al.(Marschallinger et al., 2020), Myelin-555 (25 mg/ml in PBS) was injected into the hippocampus of mice using a stereotaxic instrument (Ruiwode Life Science, China). Anesthesia was induced with isoflurane (Cat. No. R510-29, Ruiwode Life Science), and the skulls were surgically exposed. A small hole was drilled at the intended site under sterile conditions. The myelin-555 solution (1 μl) was delivered at a rate of 200 nl/min at the following coordinates: 0.7 mm lateral, −1.7 mm anterior-posterior, and −2.04 mm dorso-ventral relative to the bregma. The needle was kept in place for 5 minutes post-injection to allow for diffusion before it was slowly retracted. Postoperative care included buprenorphine for analgesia and Baytril for infection prevention. After 48 hours, mice were re-anesthetized, perfused with 4% paraformaldehyde (PFA), and the hippocampal region around the injection site was sectioned coronally into 40 μm thick slices. Sections were then stained for Iba1 and Spp1, and 5–8 sections were analyzed to quantify myelin uptake by Spp1^{+}/Iba1^{+} and Spp1^{-}/Iba1^{+} cells.

Preparation of fluorescent myelin debris for phagocytosis assays

Myelin debris was prepared from mouse brains by discontinuous sucrose-gradient ultracentrifugation (Bohlen et al., 2017; Thakurela et al., 2016). Briefly, brain homogenates in 0.32 M sucrose were layered onto 0.83 M sucrose and centrifuged at 75,000 × g for 35 min at 4°C. The 0.32/0.83 M interface was collected, washed (75,000 × g, 15 min, 4°C; then 12,000 × g, 15 min, twice), resuspended in PBS, adjusted to 100 mg/mL, and stored at −80 °C.

For fluorescent labeling, thawed myelin was pelleted (12,000 × g, 10 min, 4°C), resuspended in PBS, and incubated with Alexa Fluor 555 succinimidyl ester at a

dye:myelin ratio of 1:2 (w/w) for 30 min at room temperature in the dark. Labeled myelin was washed 3× with PBS (12,000 × g, 10 min), aliquoted, and stored at −80°C.

Cell viability

Immediately after MACS and before functional assays, CD11b+ microglia were stained with the Calcein-AM/PI Cell Viability Assay Kit (Proteintech, Cat. No.PF00007) following the manufacturer's instructions. Samples were run by flow cytometry; debris was excluded (FSC/SSC), singlets were gated, and viability was defined as Calcein-AM+ PI– (live) versus PI+ (dead). Percent viability was calculated as live/(live+dead) × 100.

JC-1 assay for mitochondrial membrane potential

BV2 cells expressing control shRNA (NC) or Spp1 shRNA (Sh-Spp1) were harvested, washed with PBS, and stained with a JC-1 probe (JC-1 Mitochondrial Membrane Potential Assay Kit, KTA4001, Abbkine, China) according to the manufacturer's instructions. After incubation at 37 °C in the dark, cells were washed, resuspended in assay buffer, and immediately analyzed on a CytoFLEX flow cytometer (Beckman Coulter, USA). JC-1 monomers and JC-1 aggregates were detected in the green and red fluorescence channels, respectively. Mitochondrial membrane potential was quantified as the JC-1 red/green fluorescence ratio (J-aggregates/J-monomers) for each sample.

Correlation Analysis

To evaluate the relationship between mitochondrial function and cognitive performance, Pearson's correlation analysis was performed using GraphPad Prism 9.0. Individual data points from both Spp1$^{fl/fl}$ and Spp1-cKO mice were pooled for the analysis. The mitochondrial OCR measured in hippocampal tissue was correlated with behavioral performance metrics, specifically the spontaneous alternation rate in the Y-maze and the time spent in the target quadrant in the Morris water maze. The Pearson correlation coefficient (r) and two-tailed P-value were calculated to assess the strength and significance of the association, with $P < 0.05$ considered statistically significant.

Supplementary Figures

Supplementary figure S1

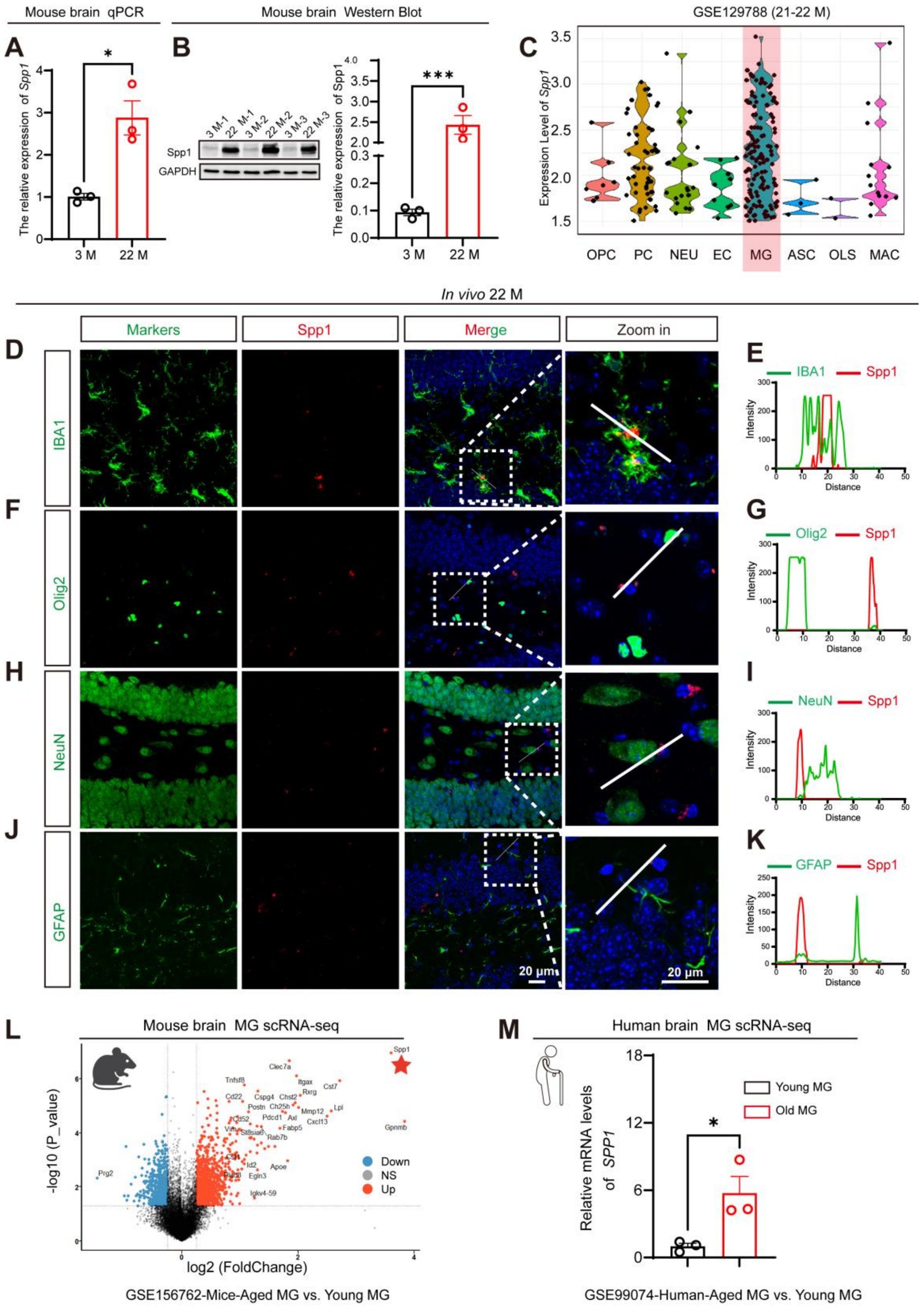

Supplementary Figure S1. Spp1 expression is significantly upregulated in the aged brain and microglia. (A–B) Quantification of *Spp1* mRNA by qPCR (A) and Spp1 protein by Western blot

(B) in the hippocampus of young (3-month-old) and aged (22-month-old) mice (n = 3 per group). (C) Violin plot showing the distribution of Spp1 expression across different cell types in the database (GSE129788) (n = 3). (D/F/H/J) Representative immunofluorescence images showing co-localization of Spp1 with specific cell markers: microglia (IBA1) (D), oligodendrocytes (Olig2) (F), neurons (NeuN) (H), and astrocytes (GFAP) (J) in brain tissue sections from aged mice. Magnified images of individual cells are indicated by dashed white boxes. Scale bars = 20 μm. (E/G/I/K) Intensity plots showing pixel intensity along the white line in the respective panels, with IBA1 (E), Olig2 (G), NeuN (I), and GFAP (K) expression in green, and Spp1 expression in red. (L) Volcano plot showing the Spp1 expression in microglia during young (6 M) and aged (23 M) mouse brain (GSE156762). (M) Bar graphs showing the Spp1 expression in microglia during adult (31–43 y; mean 38.3) and aged (84–85 y; mean 84.7) human brains (GSE99074). Data are presented as the mean ± standard error of the mean (SEM). Statistical significance was assessed using unpaired two-tailed t-tests. *$p < 0.05$, ***$p < 0.001$. Mouse pattern was created using BioRender.

Supplementary figure S2

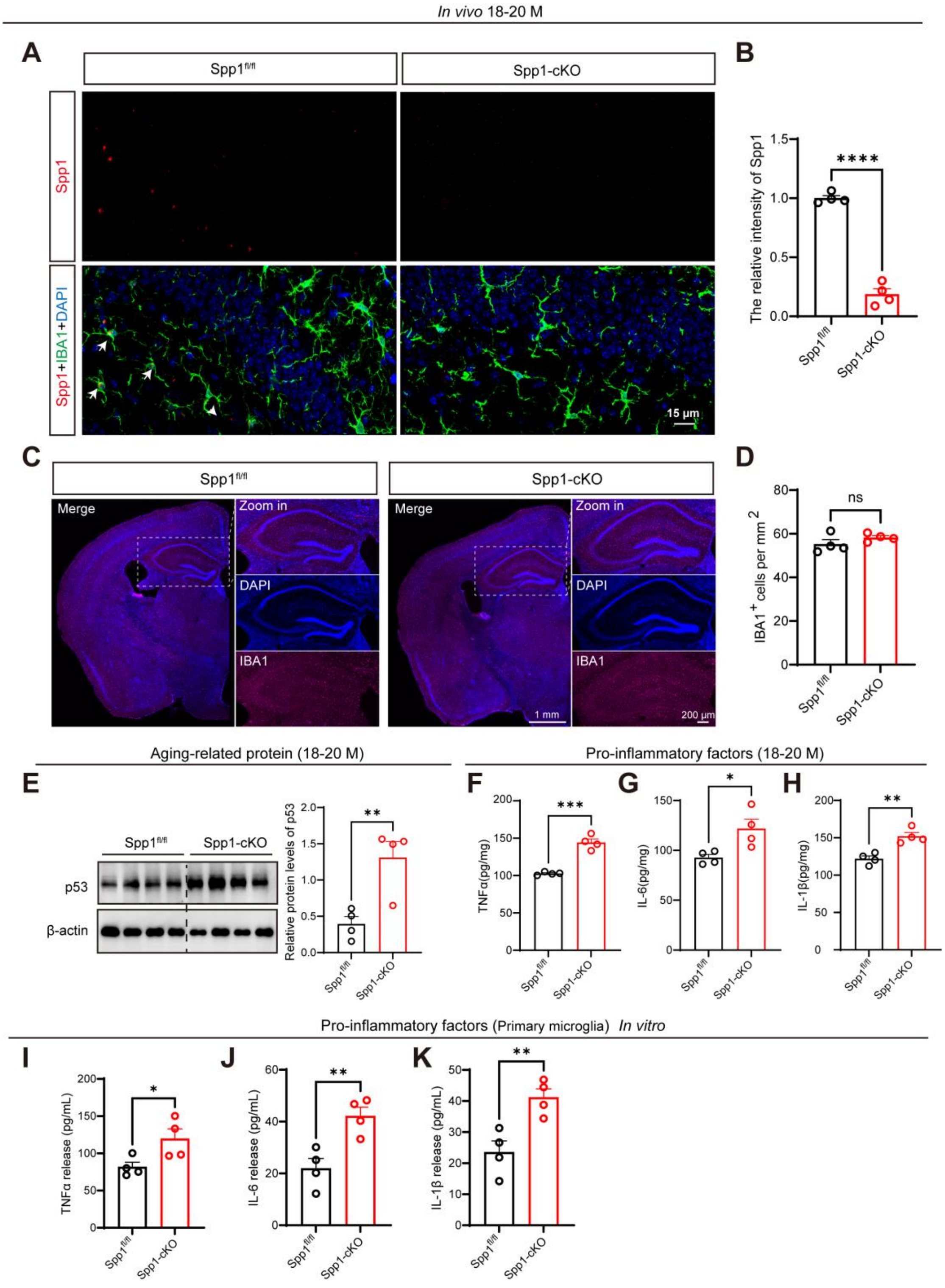

Supplementary figure S2. Microglia-specific Spp1 deletion reduces Spp1 signal but not hippocampal microglial density. (A) Representative immunofluorescence images showing Spp1 (red) and IBA1 (green) staining in brain tissue sections from Spp1$^{fl/fl}$ and Spp1-cKO mice (n = 4 per group). White arrows indicate Spp1-expressing microglia. Scale bar = 15 μm. (B) Bar graph showing the efficiency of Spp1 knockdown in microglia from Spp1-cKO mice. (C) Low-magnification views of the hippocampus with zoomed-in regions showing DAPI and IBA1 staining.

Scale bars, 1 mm (whole section) and 200 μm (zoom-in). (D) Hippocampal microglial density from $Spp1^{fl/fl}$ and Spp1-cKO mice (n = 4 per group). (E) Western blot of p53 protein expression in the hippocampal tissue of aged $Spp1^{fl/fl}$ and Spp1-cKO mice (n = 4 per group). (F-H) ELISA detected TNFα (F), IL-6 (G), and IL-1β (H) expression in the hippocampus of aged $Spp1^{fl/fl}$ and Spp1-cKO mice (n = 4 mice per group). (I-K) ELISA quantification of TNF-α (I), IL-6 (J), and IL-1β (K) levels in culture supernatants of primary microglia isolated from $Spp1^{fl/fl}$ and Spp1-cKO littermate mice (n = 4 per group). Data are presented as the mean ± standard error of the mean (SEM). Statistical significance was assessed using unpaired two-tailed t-tests. ns, no significant difference. *$p < 0.05$, **$p < 0.01$, ****$p < 0.0001$.

Supplementary figure S3

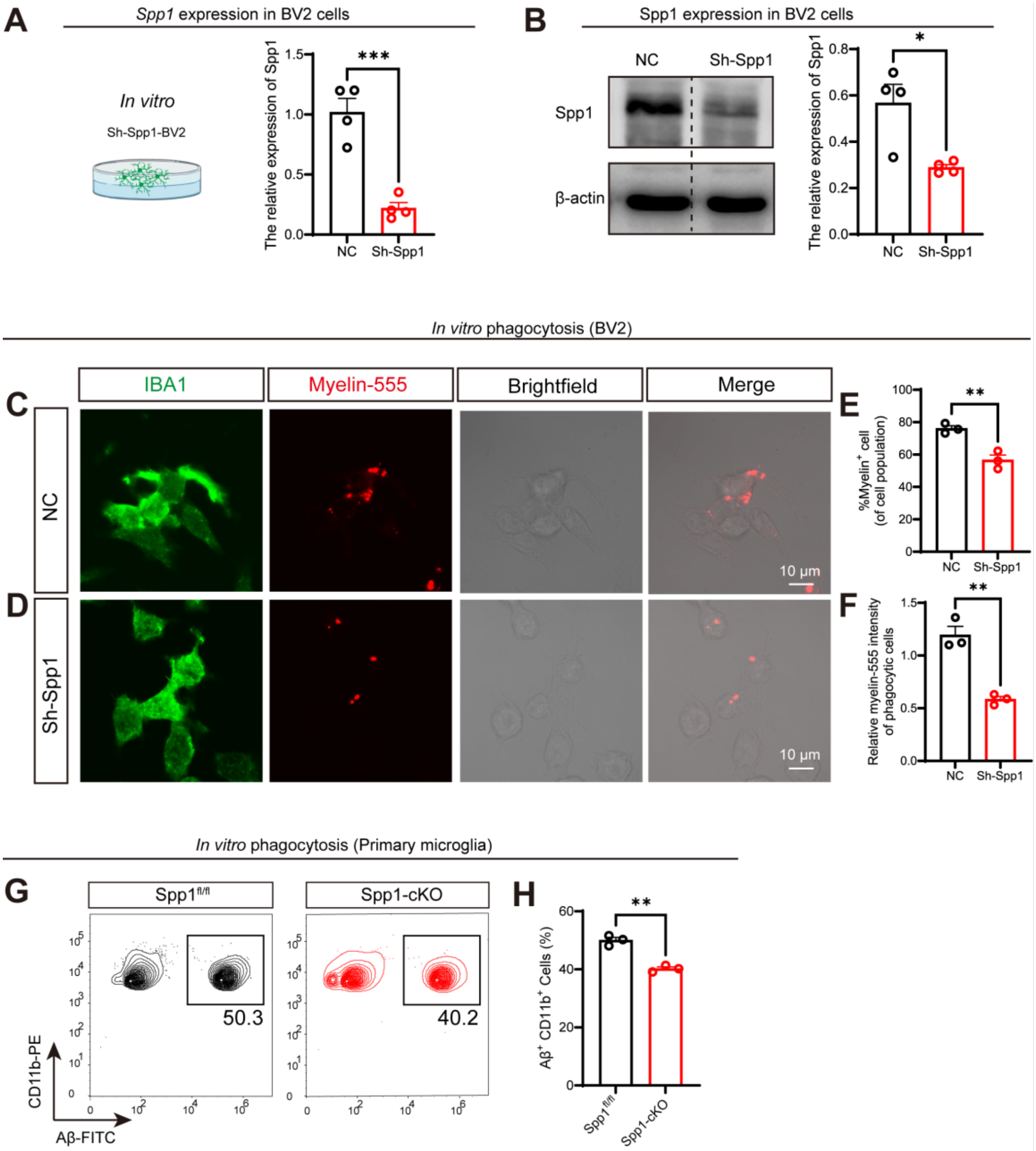

Supplementary Figure S3. Spp1 knockdown impaired microglial phagocytosis. (A) qPCR analysis of *Spp1* gene knockdownt efficiency (n = 4 per group). (B) Relative expression levels of Spp1 protein (n = 4 per group). (C-D) Representative images showing BV2 cells phagocytosing myelin-555. Scale bars = 10 μm. (E) Percentage of Myelin^{+} cells (cell population) = number of phagocytic cells/total cell count (n = 3 per group). (F) Relative myelin-555 intensity in phagocytic cells = total fluorescence intensity/total number of phagocytic cells (n = 3 per group). (G) Representative flow plots of FITC-Aβ$_{1–42}$ uptake in primary microglia from Spp1$^{fl/fl}$ and Spp1-cKO mice. (H) Quantification of the percentage of Aβ^{+} CD11b^{+} cells. (n = 3 per group). Data are presented as mean ± standard error of the mean (SEM). Groups include negative control (NC) and Spp1 gene knockdown group (Spp1-shRNA lentivirus, Sh-Spp1). Statistical significance was assessed using unpaired two-tailed t-tests. *p < 0.05, **p < 0.01. Cell pattern was created using BioRender.

Supplementary figure S4

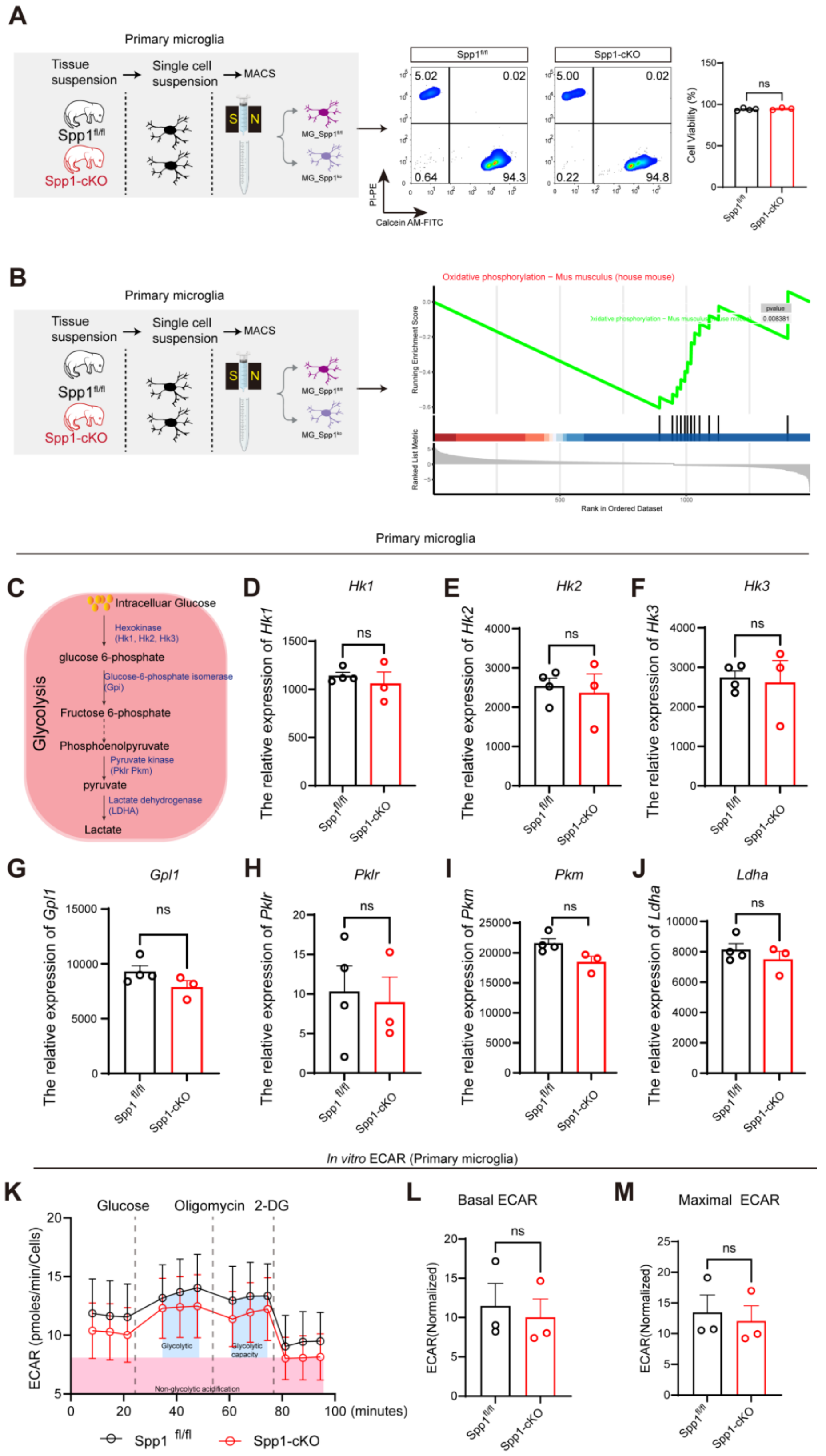

Supplementary Figure S4. Characterization of cell viability and metabolic pathways in *Spp1*-deficient primary microglia. (A) Calcein-AM/PI flow cytometry of primary microglia isolated from Spp1$^{fl/fl}$ and Spp1-cKO mice shows comparable viability (% Calcein-AM^{+} PI^{-}) after MACS.

(B) Gene Set Enrichment Analysis (GSEA) of alterations in the OXPHOS metabolic pathway in microglia (MG) between $Spp1^{fl/fl}$ and Spp1-cKO mice. (C) Key enzymes involved in the glycolysis pathway. (D-J) Changes in the expression of genes related to key glycolytic enzymes. (K) Measurement of ECAR for glycolytic capacity in primary MG from $Spp1^{fl/fl}$ and Spp1-cKO mice using Seahorse XF. (L) Basal ECAR (n = 3 per group). (M) Maximum ECAR (n = 3 per group). Data are presented as mean ± standard error of the mean (SEM). Statistical significance was assessed using unpaired two-tailed t-tests. ns, no significant difference. Mouse pattern/ Glycolytic process was created using BioRender.

Supplementary figure S5

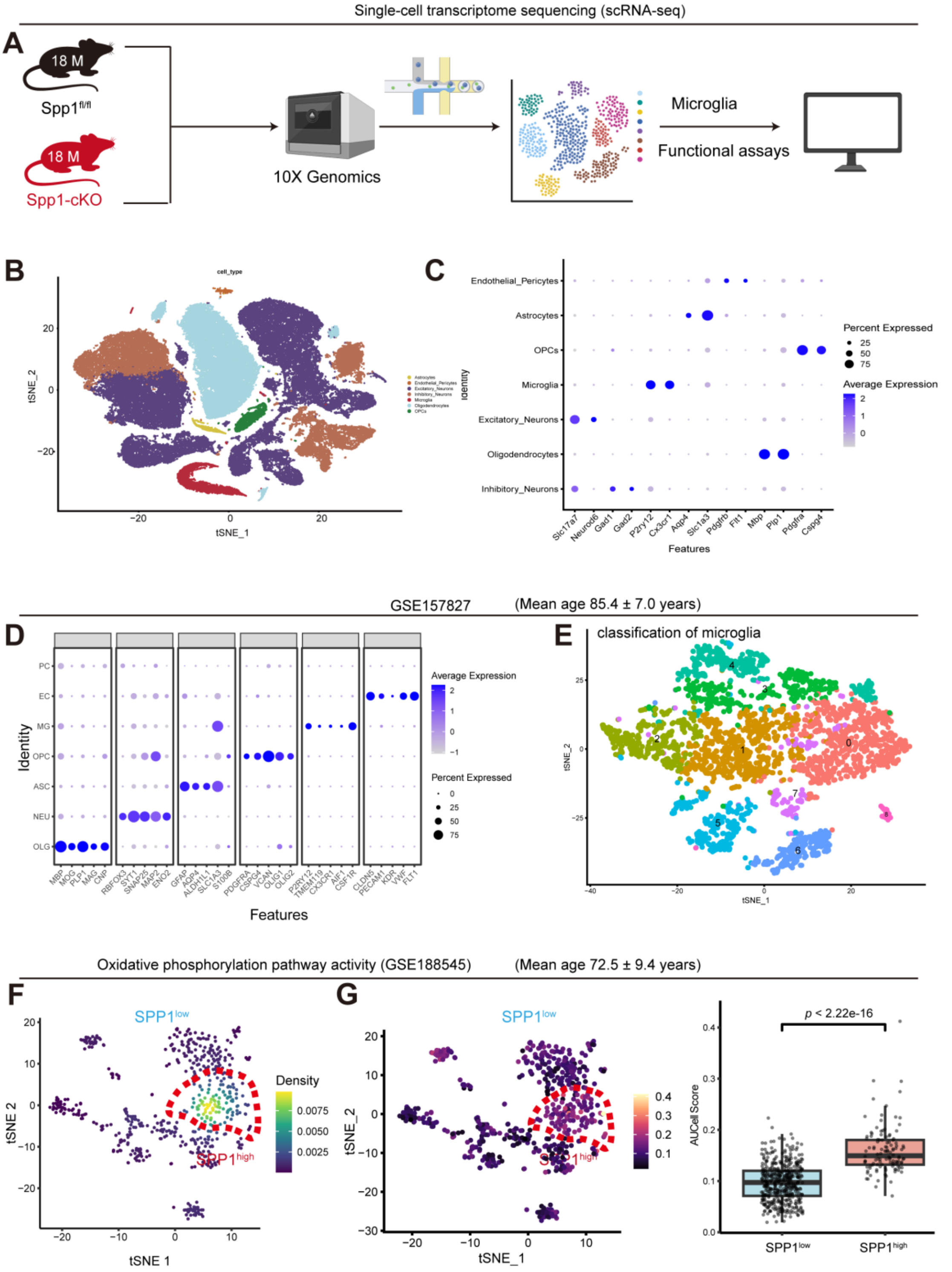

Supplementary Figure S5. Single-cell transcriptomic analysis of aged Spp1-cKO mice and validation in human aging datasets. (A) Experimental workflow for scRNA-seq of hippocampal tissue from 18-month-old mice (n=3 per group). (B–C) t-SNE plot (B) and dot plot of canonical marker genes (C) identifying major cell types, including microglia. (D) Dot plot of canonical marker genes used to annotate major brain cell types (OLG, NEU, ASC, OPC, MG, EC) in the human dataset GSE157827; dot size indicates the percentage of positive cells and color indicates average expression. (E) tSNE map of microglia (MG) from GSE157827 showing distinct microglial

subclusters in different colors. (F–G) tSNE plots of microglia from the aging human brain dataset GSE188545, showing SPP1^{low} (blue) and SPP1^{high} (red dashed circle) clusters, with color indicating cell density (F) or OXPHOS AUCell scores (G). Right, boxplot comparing OXPHOS AUCell scores between SPP1^{low} and SPP1^{high} microglia, showing significantly higher pathway activity in the SPP1^{high} population.

Supplementary figure S6

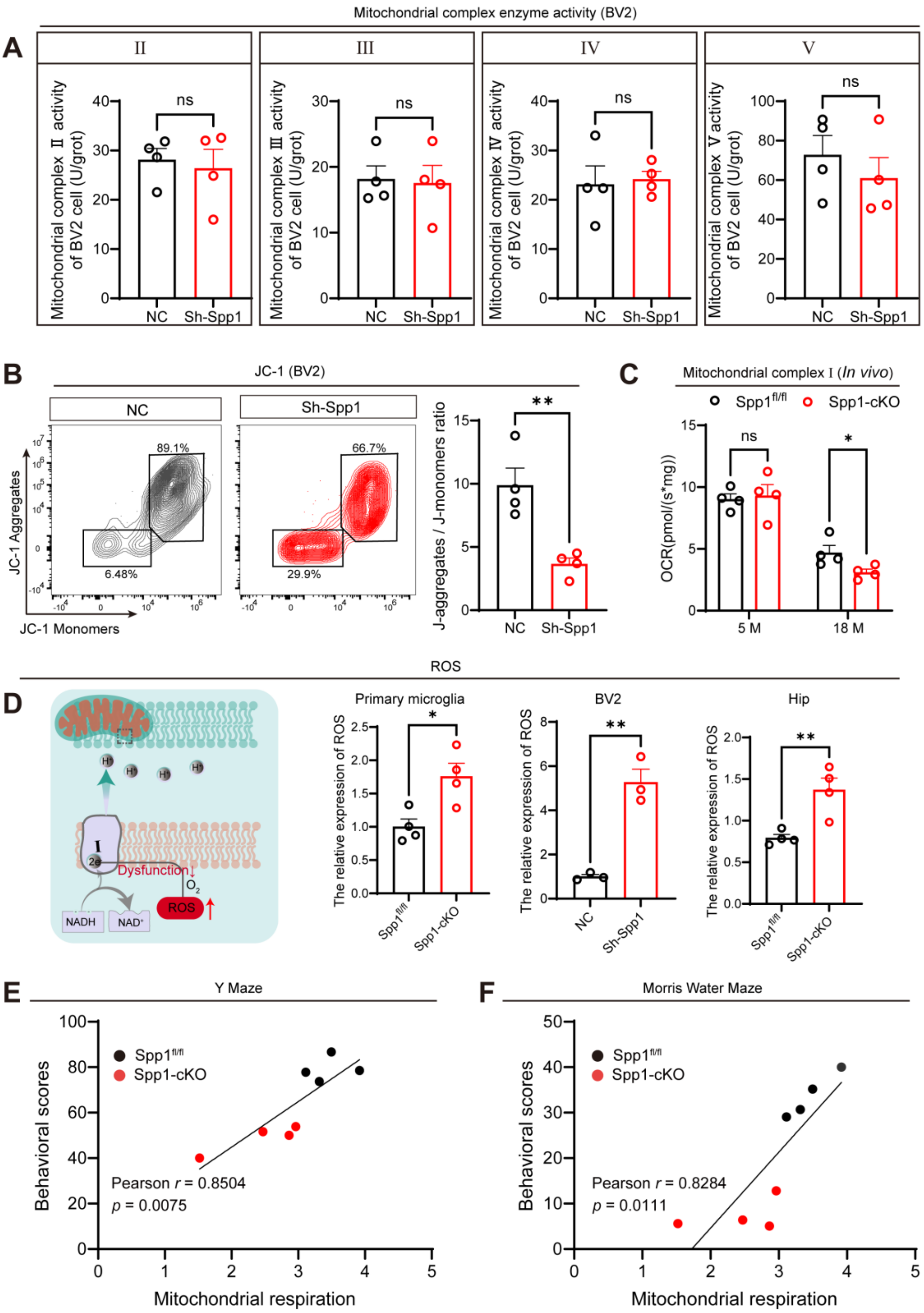

Supplementary Figure S6. Spp1 deficiency induces specific mitochondrial Complex I dysfunction and correlates with cognitive decline. (A) Enzymatic activities of complexes II–V in BV2 cells transfected with Spp1 shRNA (Sh-Spp1) and control shRNA (NC). (B) Flow cytometry analysis and quantification of JC-1 staining of BV2 cell transfected with Spp1 shRNA (Sh-Spp1) or control shRNA (NC) (n = 4). (C) Oxygen consumption rate (OCR) of complex I–linked

respiration in hippocampal mitochondria from 5-month-old (5 M) and 18-month-old (18 M) Spp1$^{fl/fl}$ and Spp1-cKO mice. (D) ROS detection results in primary microglia (*n* = 4), BV2 cells (*n* = 3), and hippocampal tissue (*n* = 4). Hip, hippocampus. (E) Pearson correlation between mitochondrial respiration and Y-maze spontaneous alternation (%). (F) Pearson correlation between mitochondrial respiration and time spent in the target quadrant in the Morris water maze probe trial. Each point represents one mouse (Spp1$^{fl/fl}$ black; Spp1-cKO, red). Data are presented as mean ± standard error of the mean (SEM). Statistical significance was assessed using unpaired two-tailed *t*-tests for (A–D) and Pearson correlation analysis for (E–F). *$p < 0.05$, **$p < 0.01$. Fig S6D of the mitochondria was created using BioRender.

Supplementary Table S1. Key Resources Table of Antibodies

REAGENT	SOURCE	IDENTIFIER/ CLONES	WB (dilution)	IF/mIHC (dilution)
Antibodies				
Rabbit anti-OPN	Zenbio	Cat. No. 340690/ Polyclonal	1:1000	1:200
Goat anti-OPN	R&D	Cat. No. AF808/ Polyclonal	1:2000	1:50
Rabbit anti-Iba-1	Wako	Cat. No. 019-197/ Polyclonal	NA	1:500
Mouse anti-GFAP	Millipore	Cat. No. MAB360/ GA5	1:1000	1:200
Rabbit anti-NeuN	Proteintech	Cat. No. 26975-1-AP/ Polyclonal	NA	1:200
Mouse anti-Tuj1	Biolegend	Cat. No. 801201/ TUJ1	1:1000	1:100
Rabbit anti-Olig2	Abcam	Cat. No. AB109186/ EPR2673	NA	1:200
Mouse anti-MBP	BioLegend	Cat. No.808401/ SMI 99	1:1000	1:100
Rat anti-Iba-1	Abcam	Cat. No. AB283346/ EPR16589	NA	1:100
Rabbit anti-β-Actin	Abclonal	Cat. No. AC038/ ARC5115-01	1:10000	NA
Rabbit anti-p53	CST	Cat. No. 2527/ 7F5	1:1000	NA
Mouse anti-pAKT	Proteintech	Cat. No. 66444-1-Ig / 1C10B8	1:5000	NA
Rabbit anti-AKT	Proteintech	Cat. No. 10176-2-AP/ Polyclonal	1:5000	NA
Rabbit anti-NDUFS2	Immunoway	Cat. No. YN0932/ Polyclonal	1:1000	NA
Donkey anti-goat (Alexa Fluor 594)	Invitrogen	Cat. No. A-11058/ Polyclonal	NA	1:1000
Donkey anti-rabbit (Alexa Fluor 488)	Invitrogen	Cat. No. A-21206/ Polyclonal	NA	1:1000
Donkey anti-goat (Alexa Fluor 488)	Jackson	Cat. No. 705-545-003/ Polyclonal	NA	1:1000
Donkey anti-rabbit (cy5)	Jackson	Cat. No. 711-175-152/ Polyclonal	NA	1:200
Donkey anti-mouse (Alexa	Invitrogen	Cat. No. A-21202/	NA	1:1000

REAGENT	SOURCE	IDENTIFIER/ CLONES	WB (dilution)	IF/mIHC (dilution)
Fluor 488)		Polyclonal		
Donkey anti-mouse (cy3)	Jackson	Cat. No. 715-165-150/ Polyclonal	NA	1:200
Donkey anti-rat (Alexa Fluor 568)	Abcam	Cat. No. ab175475/ Polyclonal	NA	1:500
Goat Anti Rabbit IgG(H+L) (HRP)	Immunoway	Cat. No. RS0002/ Polyclonal	1:5000	NA
Goat Anti Mouse IgG(H+L) (HRP)	Immunoway	Cat. No. RS0001/ Polyclonal	1:5000	NA

Supplementary Table S2. Key Resources Table of Reagents

REAGENT	SOURCE	IDENTIFIER
Bacterial and virus strains		
m-Spp1 shRNA	HANHENG	CatNo.HH20210727SXLXB-LV01
Chemicals, peptides, and recombinant proteins		
TritonX-100	Solarbio	Cat. No. IT9100
BSA	Servicebio	Cat. No. GC305010
PFA	Biosharp	Cat. No. BL539A
PBS	Servicebio	Cat. No. G4202
DMSO	Solarbio	Cat. No. D8371
FBS	Gibco	Cat. No. 26140079
RNAiso plus	TaKaRa	Cat. No. 9109
PrimeScript™ RT Reagent Kit	TaKaRa	Cat. No. RR047A
SYBR Green qPCR Master Mix	TaKaRa	Cat. No. RR820A
Neural Tissue Dissociation Kit - Papain	Miltenyi Biotec	Cat. No. 130-094-802
Isoflurane	RWD	Cat. No. R510-22
Tamoxifen	Sigma	Cat. No. 10540-29-1
Alexa Fluor™555 NHS	Thermo Scientific	Cat. No. A20009
ATP Assay Kit	Beyotime	Cat. No. S0027
ROS assay kit	Baiaolaibo	Cat. No. HR8821
ROS assay kit	Biyuntian	Cat. No. S0033M
Protein Ladder	Thermo Scientific	Cat. No. 26616
Protein Ladder	Zenbio	Cat. No. 17001
SC79	Glpbio	Cat. No. GC11645
Papain		Cat. No. LS003120
DMEM high glucose	Worthington	Cat. No. 41966052
Penicillin/streptomycin (P/S)	Gibco	Cat. No. P1400
Poly-D-lysine (PDL)	Solaibao	Cat. No. P0899
Neural Tissue Dissociation Kit	Sigma Aldrich	Cat. No. 130-092-628
gentleMACS	Miltenyi	Cat. No. 130-096-427
CD11b MicroBeads	Miltenyi	Cat. No. 130-093-634
MACS Separation Columns	Miltenyi	Cat. No. 130-042-201
	Miltenyi	
Experimental models: organisms/strains		
mouse: $Spp1^{loxp/loxp}$	Saiye Biotechno	Stock No S-CKO-05240
mouse: Cx3cr1-cre^{ERT2}	Saiye Biotechno	Stock No C001247
Software and algorithms		
GraphPad Prism 8	GraphPad	https://www.graphpad-prism.cn/
Fiji	Fiji contributors	https://imagej.net/
Rstudio	Rstudio	https://rstudio.com/products/rstudio

Supplementary Table S3. Donor characteristics and postmortem details of human hippocampal samples

	Autopsy Number	Age (years)	Sex	Clinical diagnosis	Postmortem interval (hours)	Region of Interest (ROI)
Young-1	STB011	41	Male	Amyotrophic lateral sclerosis	24h 30min	Hippocampus
Young-2	STB002	31	Male	Amyotrophic lateral sclerosis	5h 30min	Hippocampus
Young-3	Sxmu 024	43	Male	Liver cancer	8h	Hippocampus
Old-1	Sxmu 038	89	Male	Heart failure	3h	Hippocampus
Old-2	Sxmu 029	95	Male	Natural death	19h 20min	Hippocampus
Old-3	STB006	88	Male	Cardiopulmonary failure	19h 40min	Hippocampus

Supplementary Table S4. RT–qPCR primer sequences

Table S4. RT–qPCR primer sequences

Gene	Forward (5′→3′)	Reverse (5′→3′)	Reference
β-actin	CATCTTGGCCTCACTGTCCAC	GGGCCGGACTCATCGTACT	(Otkur et al., 2023)
Spp1	AGCAAGAAACTCTTCCAAGCAA	GTGAGATTCGTCAGATTCATCCG	(Zhuang et al., 2025)

References

Aibar, S., Gonzalez-Blas, C. B., Moerman, T., Huynh-Thu, V. A., Imrichova, H., Hulselmans, G., Rambow, F., Marine, J. C., Geurts, P., Aerts, J., van den Oord, J., Atak, Z. K., Wouters, J., & Aerts, S. (2017). SCENIC: single-cell regulatory network inference and clustering. *Nat Methods*, *14*(11), 1083–1086. https://doi.org/10.1038/nmeth.4463

Bohlen, C. J., Bennett, F. C., Tucker, A. F., Collins, H. Y., Mulinyawe, S. B., & Barres, B. A. (2017). Diverse Requirements for Microglial Survival, Specification, and Function Revealed by Defined-Medium Cultures. *Neuron*, *94*(4), 759–773 e758. https://doi.org/10.1016/j.neuron.2017.04.043

Chen, C., Shu, Y., Yan, C., Li, H., Huang, Z., Shen, S., Liu, C., Jiang, Y., Huang, S., Wang, Z., Mei, F., Qin, F., Liu, X., & Qiu, W. (2024). Astrocyte-derived clusterin disrupts glial physiology to obstruct remyelination in mouse models of demyelinating diseases. *Nat Commun*, *15*(1), 7791. https://doi.org/10.1038/s41467-024-52142-7

Dominguez-Lopez, S., Ahn, B., Sataranatarajan, K., Ranjit, R., Premkumar, P., Van Remmen, H., & Beckstead, M. J. (2023). Long-term methamphetamine self-administration increases mesolimbic mitochondrial oxygen consumption and decreases striatal glutathione. *Neuropharmacology*, *227*, 109436. https://doi.org/10.1016/j.neuropharm.2023.109436

Guo, H., Sun, Q., Huang, X., Wang, X., Zhang, F., Qu, W., Liu, J., Cheng, X., Zhu, Q., Yi, W., Shu, Q., & Li, X. (2024). Fucosyltransferase 8 regulates adult neurogenesis and cognition of mice by modulating the Itga6-PI3K/Akt signaling pathway. *Sci China Life Sci*, *67*(7), 1427–1440. https://doi.org/10.1007/s11427-023-2510-0

Lin, S. P., Zhu, L., Shi, H., Ye, S., Li, Q., Yin, X., Xie, Q., Xu, Q., Wei, J. X., Mei, F., Zhu, Y., Lin, P. Y., & Chen, X. H. (2023). Puerarin prevents sepsis-associated encephalopathy by regulating the AKT1 pathway in microglia. *Phytomedicine*, *121*, 155119. https://doi.org/10.1016/j.phymed.2023.155119

Mann, J., Githaka, J. M., Buckland, T. W., Yang, N., Montpetit, R., Patel, N., Li, L., Baksh, S., Godbout, R., Lemieux, H., & Goping, I. S. (2019). Non-canonical BAD activity regulates breast cancer cell and tumor growth via 14-3-3 binding and mitochondrial metabolism. *Oncogene*, *38*(18), 3325–3339. https://doi.org/10.1038/s41388-018-0673-6

Marschallinger, J., Iram, T., Zardeneta, M., Lee, S. E., Lehallier, B., Haney, M. S., Pluvinage, J. V., Mathur, V., Hahn, O., Morgens, D. W., Kim, J., Tevini, J., Felder, T. K., Wolinski, H., Bertozzi, C. R., Bassik, M. C., Aigner, L., & Wyss-Coray, T. (2020). Lipid-droplet-accumulating microglia represent a dysfunctional and proinflammatory state in the aging brain. *Nat Neurosci*, *23*(2), 194–208. https://doi.org/10.1038/s41593-019-0566-1

Otkur, W., Wang, J., Hou, T., Liu, F., Yang, R., Li, Y., Xiang, K., Pei, S., Qi, H., Lin, H., Zhou, H., Zhang, X., Piao, H. L., & Liang, X. (2023). Aminosalicylates target GPR35, partly contributing to the prevention of DSS-induced colitis. *Eur J Pharmacol*, *949*, 175719. https://doi.org/10.1016/j.ejphar.2023.175719

Thakurela, S., Garding, A., Jung, R. B., Muller, C., Goebbels, S., White, R., Werner, H. B., & Tiwari, V. K. (2016). The transcriptome of mouse central nervous system myelin. *Sci Rep*, *6*, 25828. https://doi.org/10.1038/srep25828

Wang, S., Sudan, R., Peng, V., Zhou, Y., Du, S., Yuede, C. M., Lei, T., Hou, J., Cai, Z., Cella, M., Nguyen, K., Poliani, P. L., Beatty, W. L., Chen, Y., Cao, S., Lin, K., Rodrigues, C., Ellebedy, A. H., Gilfillan, S.,…Colonna, M. (2022). TREM2 drives microglia response to amyloid-beta via

SYK-dependent and -independent pathways. *Cell*, *185*(22), 4153–4169 e4119. https://doi.org/10.1016/j.cell.2022.09.033

Zhuang, X., Zarif, M., Shen, Y., Zhang, Z., He, J., Xie, L., Wu, Q., Lin, X., Chen, K., Tian, Y., Lin, Y., Zhang, Y., Cai, Z., Qiu, Z., & Chen, L. (2025). Hepatic Abnormal Secretion of Apolipoprotein C3 Promotes Inflammation in Aortic Dissection. *J Am Heart Assoc*, *14*(3), e037172. https://doi.org/10.1161/JAHA.124.037172
